# Shifting sensitivity of septoria tritici blotch compromises field performance and yield of main fungicides in Europe

**DOI:** 10.3389/fpls.2022.1060428

**Published:** 2022-11-22

**Authors:** Lise Nistrup Jørgensen, Niels Matzen, Thies Marten Heick, Aoife O’Driscoll, Bill Clark, Katherine Waite, Jonathan Blake, Mariola Glazek, Claude Maumene, Gilles Couleaud, Bernd Rodemann, Stephan Weigand, Charlotte Bataille, Bán R, Pierre Hellin, Steven Kildea, Gerd Stammler

**Affiliations:** ^1^ Department of Agroecology, Aarhus University, Slagelse, Denmark; ^2^ NIAB, Cambridge, United Kingdom; ^3^ ADAS Rosemaund, Hereford, United Kingdom; ^4^ Institute of Plant Protection, Sosnicowice, Poland; ^5^ Arvalis Institut du végétal, Station Expérimentale, Boigneville, France; ^6^ JKI, Braunschweig, Germany; ^7^ Institut für Pflanzenschutz, Bayerische Landesanstalt für Landwirtschaft, Freising-Weihenstephan, Germany; ^8^ CRA-W, Plant and Forest Health Unit, Gembloux, Belgium; ^9^ Institute of Plant Protection, Department of Integrated Plant Protection, Hungarian University of Agriculture and Life Sciences (MATE), Gödöllő, Hungary; ^10^ Teagasc, Carlow, Ireland; ^11^ BASF Limburgerhof, Limburgerhof, Germany

**Keywords:** 14α-demethylase, SDHI fungicides, cross-resistance, fenpicoxamid, yield response, *Zymoseptoria tritici*

## Abstract

Septoria tritici blotch (STB; *Zymoseptoria tritici*) is a severe leaf disease on wheat in Northern Europe. Fungicide resistance in the populations of *Z. tritici* is increasingly challenging future control options. Twenty-five field trials were carried out in nine countries across Europe from 2019 to 2021 to investigate the efficacy of specific DMI and SDHI fungicides against STB. During the test period, two single DMIs (prothioconazole and mefentrifluconazole) and four different SDHIs (fluxapyroxad, bixafen, benzovindiflupyr and fluopyram) along with different co-formulations of DMIs and SDHIs applied at flag leaf emergence were tested. Across all countries, significant differences in azole performances against STB were seen; prothioconazole was outperformed in all countries by mefentrifluconazole. The effects also varied substantially between the SDHIs, with fluxapyroxad providing the best efficacy overall, while the performance of fluopyram was inferior to other SDHIs. In Ireland and the UK, the efficacy of SDHIs was significantly lower compared with results from continental Europe. This reduction in performances from both DMIs and SDHIs was reflected in yield responses and also linked to decreased sensitivity of *Z. tritici* isolates measured as EC_50_ values. A clear and significant gradient in EC_50_ values was seen across Europe. The lower sensitivity to SDHIs in Ireland and the UK was coincident with the prevalence of SDH-C-alterations T79N, N86S, and sporadically of H152R. The isolates’ sensitivity to SDHIs showed a clear cross-resistance between fluxapyroxad, bixafen, benzovindiflupyr and fluopyram, although the links with the latter were less apparent. Co-formulations of DMIs + SDHIs performed well in all trials conducted in 2021. Only minor differences were seen between fluxapyroxad + mefentrifluconazole and bixafen + fluopyram + prothioconazole; the combination of benzovindiflupyr + prothioconazole gave an inferior performance at some sites. Fenpicoxamid performed in line with the most effective co-formulations. This investigation shows a clear link between reduced field efficacy by solo SDHIs as a result of increasing problems with sensitivity shifting and the selection of several SDH-C mutations. The presented data stress the need to practice anti-resistance strategies to delay further erosion of fungicide efficacy.

## 1 Introduction

Severe fungal leaf disease attacks cause economically significant losses in winter wheat each year (Oerke, 2006; Savary et al., 2019). Despite efforts to mitigate these losses by employing resistant cultivars and adjusting cultural practices, frequent fungicide applications are indispensable. In Northern Europe, septoria tritici blotch (STB), caused by the ascomycete *Zymoseptoria tritici*, is one of the most devastating leaf diseases in wheat (Fones and Gurr, 2015). Besides, yellow rust (*Puccinia striiformis*) and brown rust (*Puccinia triticina*) may cause major problems depending on the region and the season (Jørgensen et al., 2014; Singh et al., 2016). Current fungicide strategies for the control of STB in most European countries are mainly built around 14α-demethylase inhibitors (DMI; FRAC group 3 and from here on referred to as azoles; e.g. prothioconazole and mefentrifluconazole), succinate dehydrogenase inhibitors (SDHI, FRAC group 11, e.g. bixafen, fluxapyroxad, and fluopyram), quinone inside inhibitors (QiI; FRAC group 21; currently only represented by the picolinamide fenpicoxamid), and multi-site fungicides (e.g. folpet and sulfur).

Over the past four decades, the intensive use of azoles in Europe has led to significant reductions in the sensitivity of contemporary European *Z. tritici* populations toward the azole fungicides (Heick et al., 2017; Blake et al., 2018; Garnault et al., 2019; Jørgensen et al., 2021a; Klink et al., 2021). The emergence of reduced sensitivity to the azoles coincided with the commercialization of novel SDHIs with activity against *Z. tritici*, starting with boscalid in 2006 and a suite of additional SDHIs from the early 2010s onwards. Since then, these SDHIs have become an integral component of disease control in cereals throughout Europe (Rehfus et al., 2018a, Turner et al., 2021). As previously seen with azoles, the increased dependency on SDHIs for control of STB has gradually led to the selection of SDHI-resistant strains of *Z. tritici*. Several key point mutations have been reported in the different *SDH* sub-units, leading to amino acid alterations in the succinate dehydrogenase (SDH) enzyme (FRAC, 2021). The most widespread of these include C-T79N and C-N86S, rendering strains moderately resistant to most SDHIs (Rehfus et al., 2020; Hellin et al., 2021). The alteration C-H152R has been identified as causing complete resistance to all the major SDHIs, and has been detected in European field *Z. tritici* populations at a low frequency; first in Ireland in 2015 and later in the United Kingdom (UK) and sporadically in Northern France, Germany, the Netherlands, and Belgium (Scalliet et al., 2012; Dooley et al., 2016; Gutiérrez-Alonso et al., 2017; Rehfus et al., 2018a; Hellin et al., 2020; FRAC, 2021; Hellin et al., 2021). Whereas the development of fungicide resistance has been problematic for the fungicide groups mentioned above, a recent study showed that target-site resistance to the newly introduced QiI fenpicoxamid was non-existing in the European *Z. tritici* population (Kildea et al., 2022). However, this active ingredient might also be at risk for resistance development, with expected increased use in the future.

The rapid development of fungicide resistance in *Z. tritici* is partially favored by its lifecycle. *Zymoseptoria tritici* causes epidemic outbreaks due to the high density and mobility of spores and widespread sexual reproduction, which allows the perpetual recombination of alleles (McDonald and Linde, 2002), followed by asexual cycles typically driven by splash events and high humidity. Therefore, anti-resistance strategies should be mandatory and followed from the introduction to keep the frequencies of resistant strains low.

The present study’s primary aim was to generate updated efficacy profiles for key azoles and SDHI fungicides commonly used for STB control in wheat across Europe. More specifically, these were: (1) to investigate the field performances of two azoles and four SDHIs against the current *Z. tritici* populations across Europe, including dose responses and efficacy of co-formulations of azoles and SDHIs, (2) to elucidate the interrelation of field performances, *in-vitro* sensitivity of *Z. tritici* populations and CYP51 and SDH-C alterations frequencies across Europe, (3) to discuss the interrelationship between EC_50_ values, specific mutations, and field performances, (4) to investigate the tested products impact on yield. This project is a continuation of a previous collaboration in the EuroWheat group – initiated by activities in the European Network of excellence - ENDURE (Jørgensen et al., 2014) and the network for comparison of azoles efficacy against STB across Europe (Jørgensen et al., 2018; Jørgensen et al., 2021a).

## 2 Methods and materials

### 2.1 Field trial setup and fungicide applications

**Figure 1:**
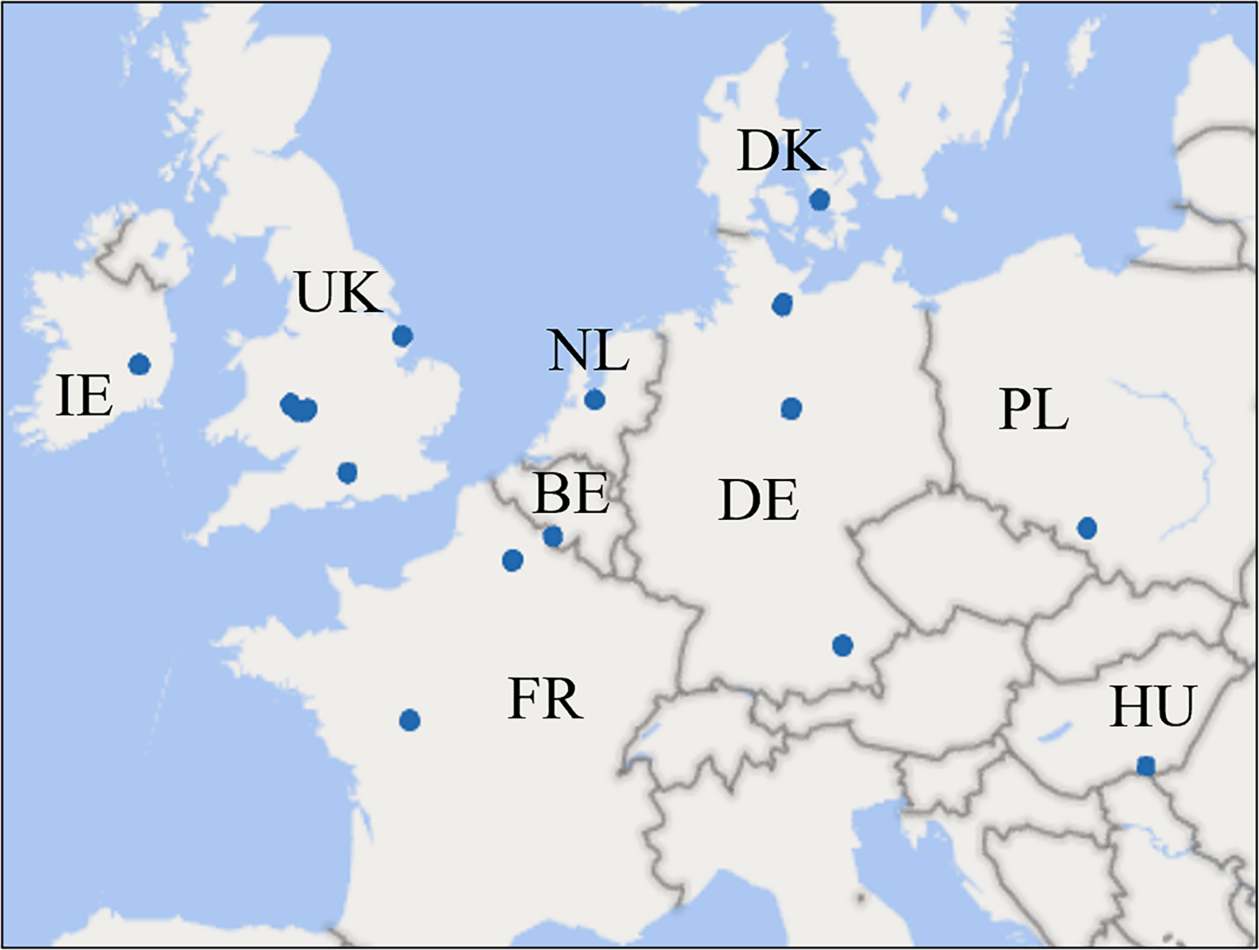
Locations of trials carried out from 2019-2021.

The study was carried out over the growing seasons of 2019, 2020, and 2021 at a range of locations across Europe, covering various climate zones and agricultural practices. In total, 33 trials were carried out across Denmark, England, Poland, France, Germany, Ireland, Belgium, the Netherlands, and Hungary by local scientific organizations (**Figure 1** and **Table S1**). The number of trials used in each analysis is presented in **Table S2**. All experiments were carried out to standard procedures using a randomized plot design with a minimum plot size of 10 m^2^ and three to four replicates at each location. Wheat varieties with moderate to high susceptibility to STB were chosen for all trials to ensure significant levels of STB. All trials have been conducted in fields with natural infections and in regions with a history of at least moderate STB attack. Except for fungicides, each trial was managed according to local agricultural practices. Fungicides were applied using various equipment, including knapsack sprayers and self-propelled sprayers, depending on local options, with both water volumes (196 – 300 L/ha) used and pressures (1.8 – 4 bar) applied reflective of local equipment and practices. All applications were made to coincide with flag leaf emergence at the growth stage (GS) 37-39 (BBCH) (Lancashire et al., 1991). In a few cases, the trials were treated with cover sprays to mitigate early attacks of powdery mildew, yellow rust, and STB as required.

### 2.2 Fungicide application scheme

Over the three seasons, a total of four different trial protocols were used, but as commonalities existed between them, it is possible to examine three different factors a) comparisons between key solo azoles and SDHIs (**Table 1**); b) comparisons between full and half rates for selected azoles and SDHIs (**Table 1**); c) comparisons of azole/SDHI mixtures and the novel QiI fenpicoxamid (**Table 2**). The objective and specific details of each are further outlined below. The fungicides used in all trials were supplied by BASF SE (Limburgerhof, Germany).

**Table 1:** Treatments used in all trials with full dose applications were carried out at growth stage GS 37-39.

Treatments	Active ingredient	Abbreviation	Active group	Years	Dose (l/ha)	g a.i./ha
Untreated control	-				-	-
Revysol, BASF	Mefentrifluconazole	MFA	DMI	2019, 2020, 2021	1.5	150
Proline 250, Bayer	Prothioconazole	PTH	DMI	2019, 2020, 2021	0.8	200
Imtrex, BASF	Fluxapyroxad	FXP	SDHI	2019, 2020, 2021	2	125
Thore, Bayer	Bixafen	BIX	SDHI	2019, 2020, 2021	1	125
Elatus, Syngenta	Benzovindiflupyr	BVF	SDHI	2019, 2020, 2021	0.75	75
Luna, Bayer	Fluopyram	FLP	SDHI	2019, 2020	0.2	100

In 2019, also half rates were tested for the included actives.

In 2019, also half rates were tested.

**Table 2:** Treatment mixtures and fenpicoxamid at full label doses from trials carried out in 2021.

Treatments	Active ingredient	Abbreviation	Active group	Dose (l/ha)	g a.i./ha
Untreated control	-			-	-
Questar, Corteva	Fenpicoxamid	FPX	QiI	2.0/1.5*	100/75
Revystar XL, BASF	Fluxapyroxad+ mefentrifluconazole	FXP+MFA	SDHI+DMI	1.5	75 +150
Revytrex, BASF	Fluxapyroxad+ mefentrifluconazole	FXP+MFA	SDHI+DMI	1.5	100 + 100
Elatus Era, Syngenta	Benzovindiflupyr+ prothioconazole	BVF+PTH	SDHI+DMI	1.0	75 + 150
Ascra Xpro, Bayer	Bixafen+fluopyram +prothioconazole	BIX+FLP +PTH	SDHI+SDHI +DMI	1.5	98 + 98 + 195
Silvron Xpro, Bayer	Bixafen+fluopyram	BIX+FLP	SDHI+SDHI	1	100 + 100

*The lower dose was used in FR and BE in accordance with the authorized rate in these countries.

*a) Azole & SDHI comparisons*

In each of the three seasons, the efficacy of the azoles mefentrifluconazole (MFA) and prothioconazole (PTH), and the SDHIs fluxapyroxad (FXP), bixafen (BIX) and benzovindiflupyr (BVF) were investigated as solo products, with fluopyram (FLP) included in only 2019 and 2020 (**Table 1**).

*b) Comparisons between full and half doses*

In 2019, treatments with the solo azoles MFA and PTH and the solo SDHIs FXP, BVF, BIX, and FLP were tested at half rates (**Table 1**) in addition to the full rates.

*c) Azole/SDHI mixtures & QiI fenpicoxamid*

In 2021, comparisons between full label rate treatments were made using the co-formulations of the azoles and SDHIs FXP+MFA, BVF+PTH, and BIX+FLP+PTH, the SDHIs BIX+FLP and the QiI fenpicoxamid (FPX) (**Table 2**).

### 2.3 Field assessments

The severity of STB was assessed following EPPO guidelines (1/26 (4)) (Oepp/Eppo, 2014). Assessments were carried out individually on the flag leaf (F) and flag leaf minus one (F-1), by visually scoring the average percent leaf area with symptoms either at four different spots in each plot or on ten main stems selected from throughout the plot. The assessment carried out at GS 73-75 was emphasized, corresponding to 27-55 days after application (DAA). The analyzed assessments on F-1 were generally carried out at an earlier timepoint than those on flag leaves. All trials were carried through to harvest, and grain yields were measured for every plot and adjusted to 85% dry matter.

### 2.4 *Zymoseptoria tritici* fungicide sensitivity testing and molecular analyses

Twenty leaves with STB symptoms were collected per replicate from the fungicide untreated plots at GS 75 from every trial. Ten leaves were sent to BASF SE (Limburgerhof, Germany) for analysis of alterations associated with azole and SDHI resistance (pyrosequencing and qPCR) and ten were sent to EpiLogic (Freising, Germany) for fungicide sensitivity testing (microtitre assessment and EC_50_ calculations). To determine the frequency of key alteration associated with azole and SDHI resistance, leaves from each of the four replicates of individual trials were pooled into one bulk sample of 40 leaves. All diseased material from the pooled- leaves was collected in one sample (15 to 30 mg), which was ground for 1 min at 20 hz (Retsch) in the presence of a metal bead. Genomic DNA was extracted using NucleoSpin Plant II kit (Macherey-Nagel) following the manufacturer’s protocol. Mutation analysis was carried out using a combination of pyrosequencing or qPCR (Rehfus, 2018b; Huf, 2020). The specific mutations investigated were SDH-B alterations N225I, N225T, H267X, T268I, and I269V, SDH-C alterations T79I, T79N, W80S, N86S and H152R in all three seasons, G90R in 2019 and 2020 and S83G in 2021, and CYP51 alterations D134G, V136A, V136C, A379G, I381V and S524T in all three seasons. In the case of samples analyzed for CYP51 mutations in 2019 and sensitivity to PTH-D, five trials were located away from the trial site where efficacy were measured, but both are expected to reflect their national level of sensitivity in 2019 (**Table S1**).

To determine the sensitivity of the local *Z. tritici* population in each trial to the SDHI and azole fungicides, ten single pycnidium isolates were retrieved from the other 40 leaves collected across the fungicide untreated plots in most cases (**Table S3**). Sensitivity was determined using a microtitre plate assay as previously described by Rehfus et al. (2018), with the SDHI fungicides FXP, BIX, FLP, and BVF and azole prothioconazole-desthio (PTH-D) tested each year and the azole MFA tested in only 2020 and 2021. The range of fungicide concentrations included differed depending on the fungicide (**Table 1**). No sensitivity data was generated for three trials in the United Kingdom, the Netherlands, and Germany in 2019 and one trial in Hungary in 2021, as no disease was present.

### 2.5 Statistical analyses

Statistical analyses were carried out using RStudio version 09.2 + 382 (RStudio, 2021) with α = 0.05 for all tests. The data were checked for normal distribution using QQ-plots and the Shapiro-Wilk normality test. Furthermore, residual plots and Bartlett’s test were used to check for homogeneity of variance. However, it was not possible to correct the residuals by a transformation of variables and removal of outliers. Thus, all analyses were carried out using the non-parametric Kruskal-Wallis test and differences between groups were distinguished using Dunn’s test, using the appropriate functions from the FSA R package (Ogle et al., 2022). For the correlation analysis, Pearson’s r correlation coefficients and probability values were calculated using *corr.test* of the “psych” package. Holm’s correction was used to adjust for multiple comparisons.

## 3 Results

### 3.1 Overall disease pressures

**Figure 2:**
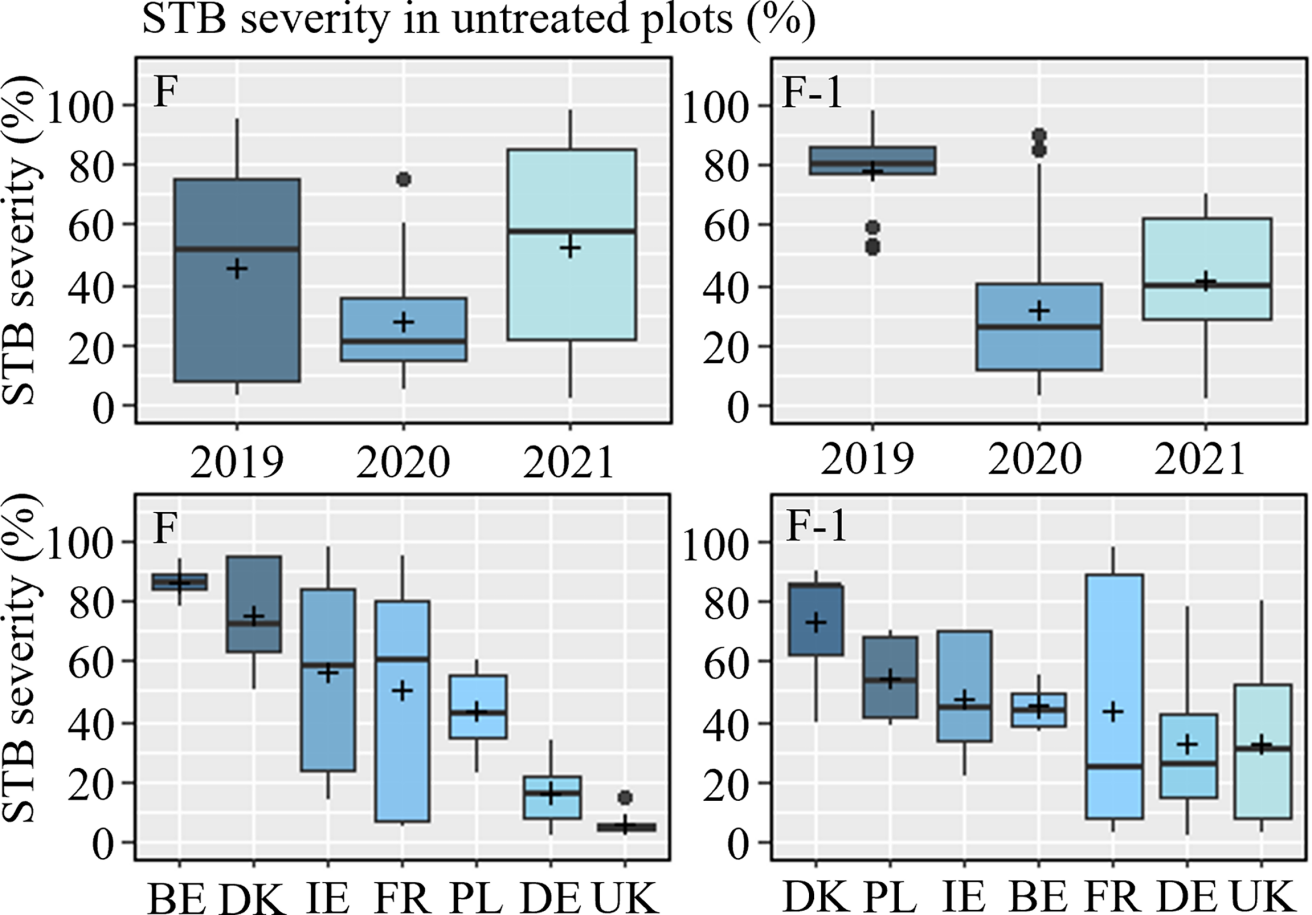
Septoria tritici blotch (STB) severity in untreated plots (%) on flag leaves (F) (left) and flag leaf minus one (F-1) (right) from 2019-2021, sorted by year (top) and country (bottom). The line inside the boxes indicates the median and ‘+’ indicates the average.

Of the 25 trials included in the STB dataset, which were conducted over three seasons, a total of 18 and 19 trials developed adequate levels of STB on flag leaves (F) and F-1, respectively. Disease severity above 4% was considered a minimum for inclusion in the dataset. Disease pressures varied across seasons, with the highest pressures observed in 2019, followed by 2021, with the lowest levels recorded in 2020, which was regarded as a low-pressure season across much of Europe (**Figure 2** and **Table S4**). Similarly, disease levels varied between countries, with the highest levels observed in Belgium (albeit it was only included in 2021) and Denmark, moderate levels in Germany, Ireland and Poland, and low levels in the UK (**Figure 2**). Across the three seasons, levels of STB in France varied considerably.

### 3.2 Fungicide efficacies

*a) Azole & SDHI comparisons*

**Figure 3:**
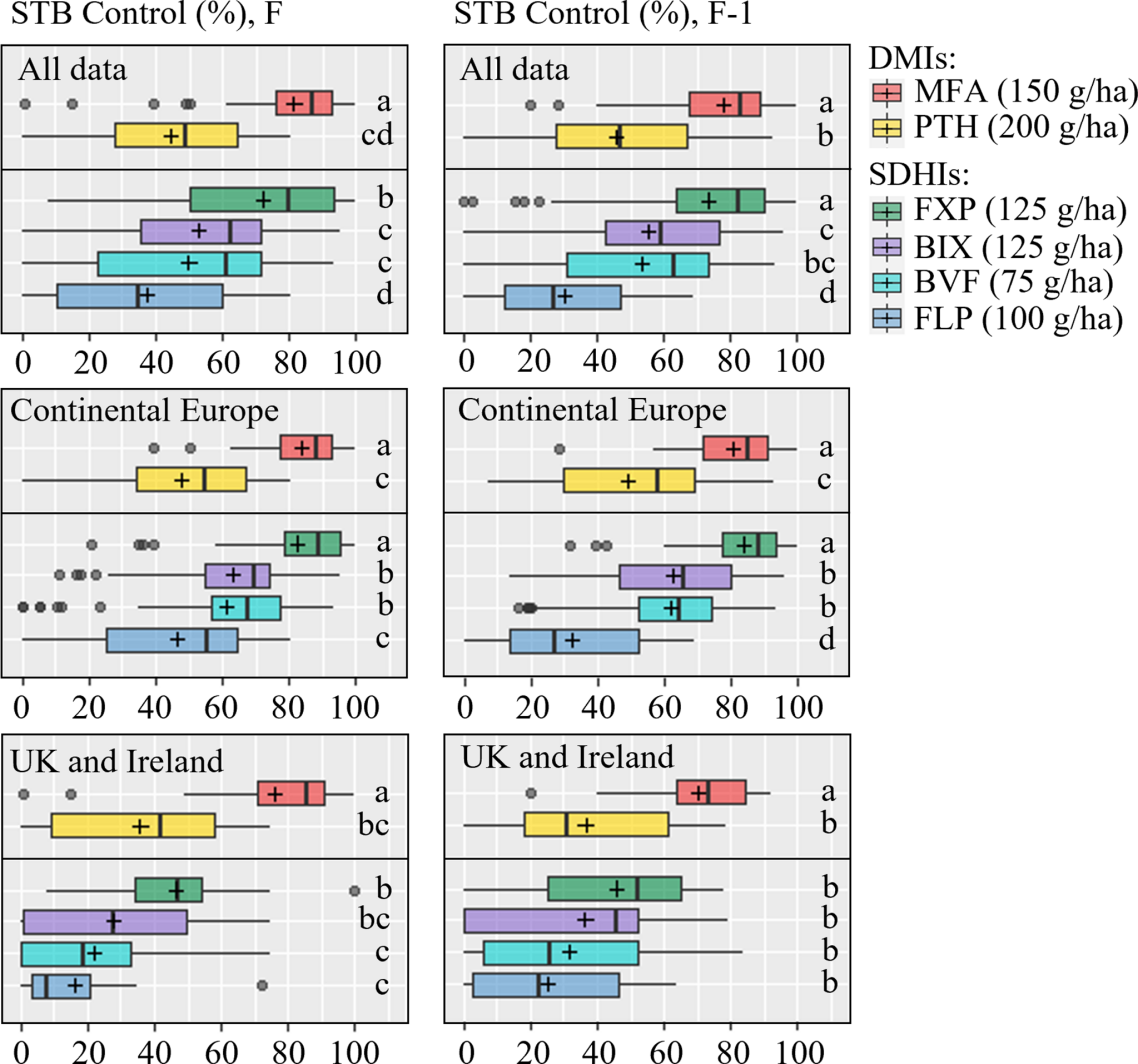
Septoria tritici blotch (STB) control (%) on flag leaves (left) and F-1 (right) were assessed at growth stage 65-85, 22-58 days after application in 18 trials on flag leaf and 19 on F-1 from 2019-2021. Trials from Continental Europe were carried out in Denmark, France, Germany, Poland and Belgium. Tested fungicides included mefentrifluconazole (MFA), prothioconazole (PTH), fluxapyroxad (FXP), bixafen (BIX), benzovindiflupyr (BVF), fluopyram (FLP). FLP was not included in 2021. The line inside the boxes indicates the median and ‘+’ indicates the average. Different letters represent significant differences between treatments within boxplot as determined by Dunn’s test.

Although levels of control provided by the individual solo treatments when applied at the full recommended field rate varied considerably between locations, a pattern of control could be observed. On the flag leaf, the azole MFA provided the most control, followed by the SDHI FXP, which was superior to the SDHIs BIX and BVF, both of which provided only moderate levels of control comparable to the azole PTH, but significantly more when compared to the SDHI FLP (**Table 3** and **Figure 3**). The variations in levels of control from the different treatments were similar across all trials (**Figure 3**), with standard deviations ranging from 24.5-30.7% for PTH, FXP, FLP, BIX and BVF on flag leaves, while the level of control from MFA was least variable (sd=18.1%). The efficacy patterns were similar on F-1, but at moderately lower levels, than those seen on flag leaves (**Table 4** and **Figure 3**).

**Table 3:** Overview of septoria tritici blotch (STB) control and disease severity (%) in untreated (untr.) plots on flag leaves.

STB control (%), flag leaf					Untr.	FXP	BIX	BVF	FLP	PTH	MFA
Year	Trial	Ctry.	GS	DAA		125 g/ha	125 g/ha	75 g/ha	100 g/ha	200 g/ha	150 g/ha
2019	19309-1	DK	75	43	95.0	88	68	17	9	11	84
	19309-3	UK	75	40	4.1	36	13	17	13	13	41
	19309-4	IE	75	28	58.3	39	47	38	15	25	76
	19309-6	FR	75	55	65.9	97	70	86	34	34	94
	19309-7	DE	73	30	7.7	100	69	61	26	70	79
2020	20334-1	DK	75	42	61.3	96	93	92	72	58	97
	20334-3	DE	75-77	43	17.5	93	69	84	66	69	93
	20334-4	FR	85	58	6.2	90	74	69	56	76	92
	20334-5	PL	75	48	31.3	80	72	62	61	61	90
	20334-8	IE	75	49	18.6	42	11	25	21	39	73
2021	21328-1	DK	77	43	70.0	81	73	73	–	36	65
	21328-3	UK	75	38	8.0	72	50	22	–	50	100
	21328-4	IE	75	51	91.3	42	20	9	–	51	90
	21328-5	BE	75	38	86.1	65	25	30	–	29	82
	21328-6	FR	75	48	92.5	75	58	70	–	66	87
	21328-7	PL	75	45	55.0	81	61	73	–	39	75
	21328-9	DE	75	30	29.2	90	58	62	–	60	91
	21328-10	DE	83	40	10.7	44	33	28	–	28	65
Avg., 2019					45.2	70.7	52.4	41.6	18.9	30.2	73.9
Avg., 2020					28.1	79.5	63.1	66.3	55.2	59.8	89.0
Avg., 2021					52.9	68.5	46.9	45.1	–	43.4	81.7
Avg., 2019-2021					43.8	72.1	52.9	50.0	37.5	44.3	81.5
Avg., continental Europe, 2019-2021					47.0	82.7	63.0	61.3	46.5	47.9	83.8
Avg., Ireland, and UK, 2019-2021					36.1	46.2	28.0	22.1	16.4	35.8	76.0

STB severity was assessed at growth stage (GS) 73-85, 30-58 days after application (DAA) in 18 trials from 2019-2021. Trials from Continental Europe were carried out in Denmark (DK), France (FR), Germany (DE), Poland (PL) and Belgium (BE). Tested fungicides included mefentrifluconazole (MFA), prothioconazole (PTH), fluxapyroxad (FXP), bixafen (BIX), benzovindiflupyr (BVF), fluopyram (FLP). Colors signify ranking of treatment effects within individual trials. Efficacy data was ranked using a color gradient for each individual trial; the ranking should therefore be read horizontally and not vertically. Green: highest rating. Yellow: medium rating. Orange: lowest rating.

**Table 4:** Overview of septoria tritici blotch (STB) control and disease severity (%) in untreated (untr.) plots on flag leaves minus one (F-1).

STB control (%), flag leaf -1					Untr.	FXP	BIX	BVF	FLP	PTH	MFA
Year	Trial	Ctry.	GS	DAA		125 g/ha	125 g/ha	75 g/ha	100 g/ha	200 g/ha	150 g/ha
2019	19309-1	DK	73	33	86.3	86	62	42	20	20	83
	19309-3	UK	75	40	61.1	23	12	8	11	11	50
	19309-6	FR	75	55	92.5	83	43	56	8	13	72
	19309-7	DE	73	30	77.1	90	28	19	12	30	59
2020	20334-1	DK	75	42	85.0	76	77	69	22	18	89
	20334-2	DE	75	37	27.3	52	40	43	25	33	66
	20334-3	DE	75-77	43	17.5	84	56	74	56	63	77
	20334-4	FR	75	44	6.3	93	48	65	44	68	79
	20334-5	PL	75	48	40.5	80	80	68	67	72	92
	20334-6	UK	78	43	5.7	32	35	17	16	23	72
	20334-8	IE	75	49	32.3	49	34	43	49	54	82
2021	21328-1	DK	75	35	47.5	94	92	93	–	50	89
	21328-2	UK	69	41	30.4	69	53	61	–	54	79
	21328-4	IE	65	35	63.3	55	48	30	–	41	69
	21328-5	BE	75	27	45.0	93	64	70	–	64	91
	21328-6	FR	71	33	24.6	97	94	90	–	84	93
	21328-7	PL	71	30	68.3	98	76	70	–	63	89
	21328-9	DE	69	22	36.4	89	72	68	–	55	90
	21328-10	DE	70	27	5.6	62	43	46	–	60	61
Avg., 2019					78.4	69.6	35.9	29.5	13.3	18.9	65.6
Avg., 2020					31.5	65.7	52.9	53.7	39.7	46.5	79.6
Avg., 2021					41.2	81.7	67.0	65.4	–	57.9	82.2
Avg., 2019-2021					45.3	73.3	55.4	53.7	30.3	45.7	77.8
Avg., Continental Europe, 2019-2021					47.9	83.7	62.6	62.0	32.2	49.1	80.6
Avg., Ireland + UK, 2019-2021					38.6	45.7	36.4	31.8	25.4	36.5	70.4

STB severity was assessed at growth stage (GS) 69-78, 22-55 days after application (DAA) in 19 trials from 2019-2021. Trials from Continental (Cont.) Europe were carried out in Denmark (DK), France (FR), Germany (DE), Poland (PL) and Belgium (BE). Tested fungicides included mefentrifluconazole (MFA), prothioconazole (PTH), fluxapyroxad (FXP), bixafen (BIX), benzovindiflupyr (BVF), fluopyram (FLP). Colors signify ranking of treatment effects within individual trials. Efficacy data were ranked using a color gradient for each individual trial; the ranking should therefore be read horizontally and not vertically. Green: highest rating. Yellow: medium rating. Orange: lowest rating.

As significant differences have previously been observed in the frequencies of key alterations affecting both the azoles and SDHIs between Ireland and the UK with the rest of Europe (Hellin et al., 2021), levels of efficacy were separately analyzed for Ireland and the UK, and Continental Europe (Belgium, Denmark, France, Germany, Poland). In Ireland and the UK, on the flag leaf, the azole MFA provided the best control (76%), which was significantly greater than any of the other fungicides. FXP only provided moderate levels of disease control (46%) and was not significantly better than either the SDHI BIX or the azole PTH, both of which did not provide significantly better control than the remaining SDHIs BVF or FLP. On F-1, the only differences observed were between MFA and all other fungicides, with MFA providing superior control (70%). This contrasts with Continental Europe, where on the flag leaf the SDHI FXP provided equal high levels of disease control (83%) to MFA (84%), which were significantly better than the good levels of control provided by BIX (63%) and BVF (61%), which were also significantly better than levels provided by either PTH (48%) or FLP (47%). Similar levels of control and differences were observed on F-1, with the exception that the difference between PTH and FLP was significant, with PTH providing better disease control.

*b) Comparisons between full and half doses*

**Figure 4:**
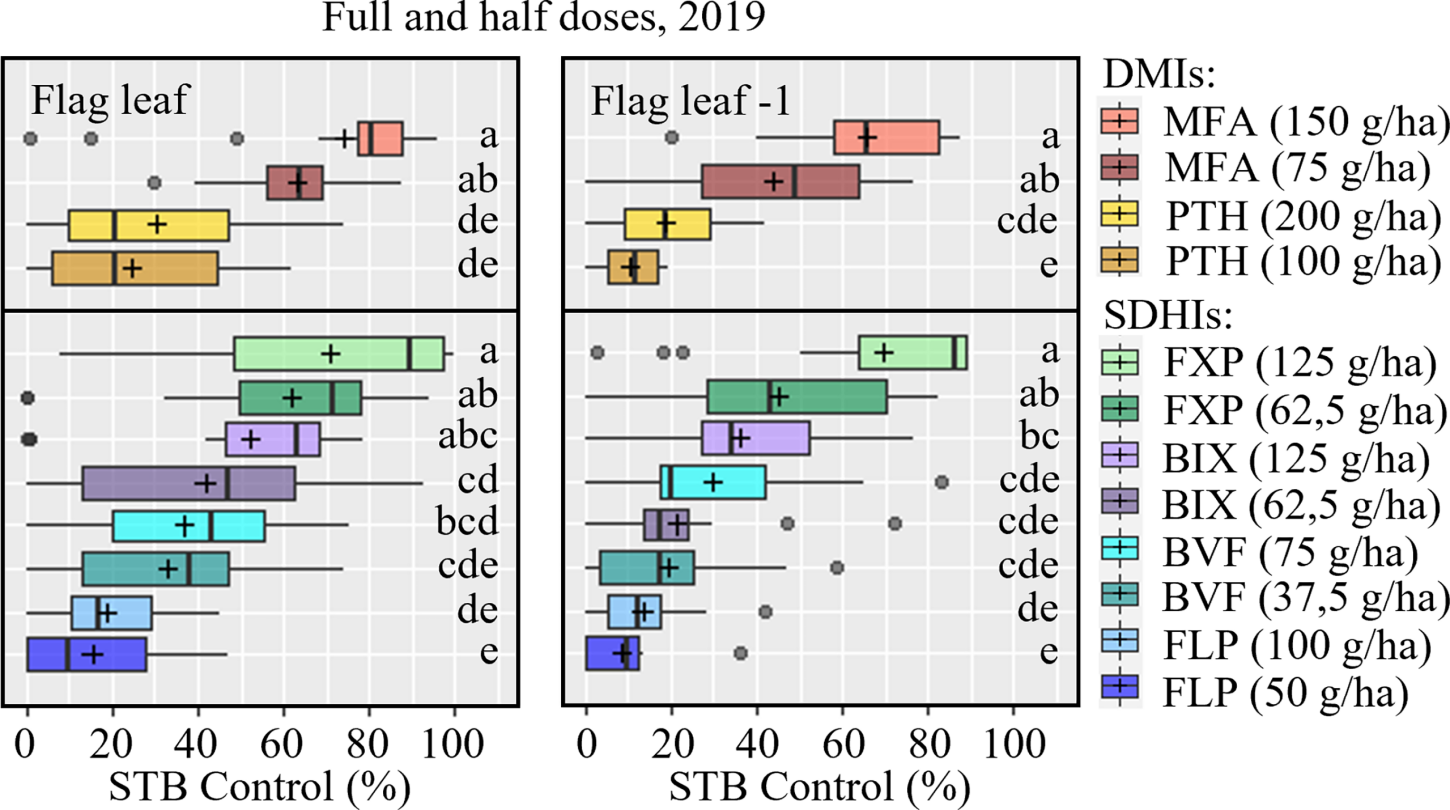
STB control (%) of full and half doses on flag leaves (left) and F-1 leaves (right) were assessed at growth stage 73-75, 33-55 days after application in five trials in 2019. The trials were carried out in Denmark, the United Kingdom, Ireland, France, and Germany. The tested fungicides included mefentrifluconazole (MFA), prothioconazole (PTH), fluxapyroxad (FXP), bixafen (BIX), benzovindiflupyr (BVF), fluopyram (FLP). The line inside the boxes indicates the median and ‘+’ indicates the average. Different letters represent significant differences between treatments within boxplot as determined by Dunn’s test.

Full and half doses were tested in 2019, and usable data on the effects against STB were collected from five trials (DK, UK, IE, FR, DE) (**Table S1**). The full dose of every compound tended to provide better STB control than the half doses; this was most pronounced in the case of FXP and MFA (**Figure 4** and **Table S5**). The exception to this dose-response was in Ireland where FXP, FLP and PTH had lower efficacies at full dose than at half dose. On F-1, a similar overall pattern in efficacy was observed, although the difference between full and half rate MFA was not significantly different.

*c) Azole/SDHI mixtures & QiI fenpicoxamid*

**Figure 5:**
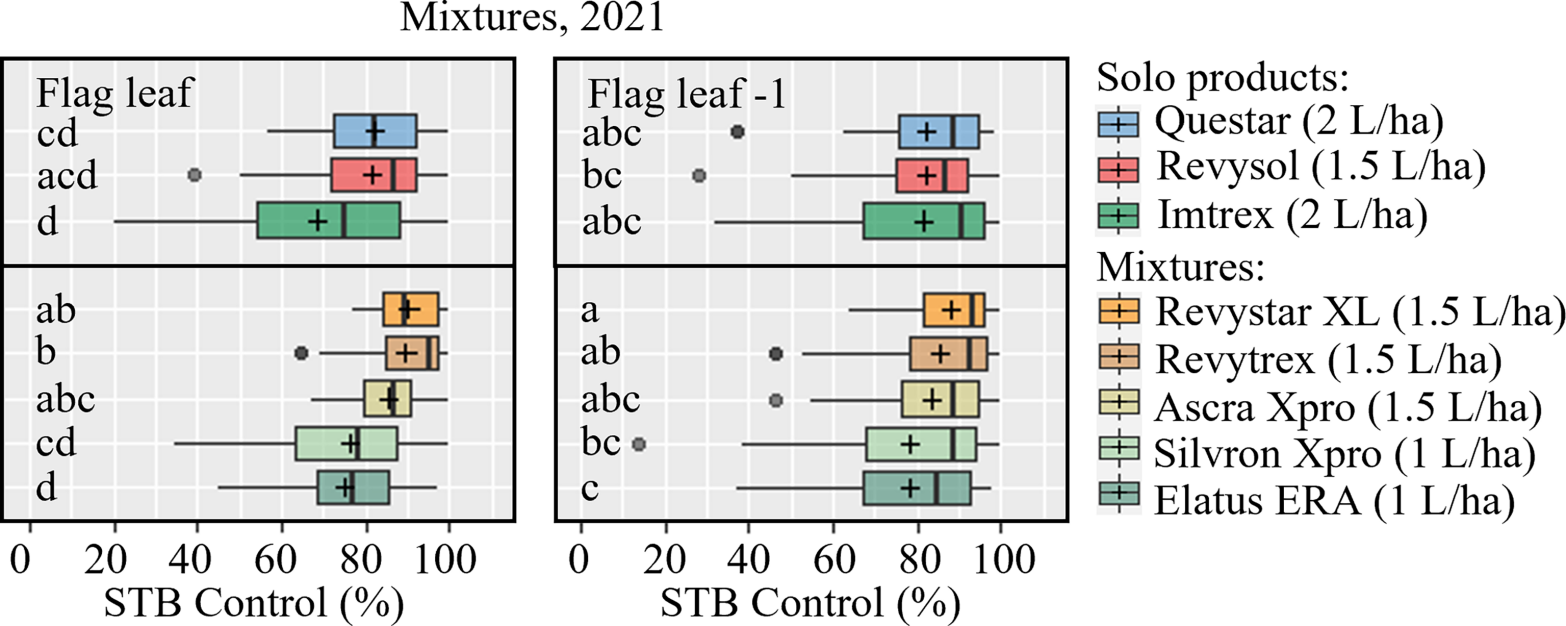
STB control (%) of mixtures on flag leaves (left) and F-1 leaves (right) were assessed at growth stage 65-83, 27-51 days after application in 9 trials in 2021. The trials were carried out in Denmark, the United Kingdom, Ireland, Belgium, France, Poland, and Germany. The tested fungicides included Questar (fenpicoxamid), Revysol (mefentrifluconazole), Imtrex (fluxapyroxad), Revystar and Revytrex (fluxapyroxad + mefentrifluconazole), Ascra Xpro (bixafen + fluopyram + prothioconazole), Silvron Xpro (bixafen + fluopyram), Elatus ERA (benzovindiflupyr + prothioconazole). The line inside the boxes indicates the median and ‘+’ indicates the average. Different letters represent significant differences between treatments within boxplot as determined by Dunn’s test.

During the 2021 trial season five commercial co-formulations were tested in 10 trials, of which 9 provided useable data (**Table 5**). Four of the co-formulations were based on the combination of azole + SDHI(s). For comparison, one co-formulation containing two SDHIs (Silvron Xpro) was tested and compared with the most potent solo SDHI (FXP in the form of Imtrex), solo azole (MFA, in the form of Revysol), and the new QiI fenpicoxamid (in the form of Questar). This testing clearly showed a stable and improved control of the co-formulations compared with either the azole or the SDHI used alone (**Table 5**). While MFA solo provided on average 72% and 76% control on F-1 and flag leaf, respectively; FXP similarly gave 69% and 82% control. Elatus ERA and Silvron Xpro gave comparable control to solo FXP, while both Revystar XL, Revytrex, Ascra Xpro and the new QiI fungicide Questar gave the highest average control. On F-1, the effects of the different solutions were overall very similar (**Figure 5**). If looking specifically at the UK and Irish trial sites, the efficacies from the solo SDHIs were clearly lower compared with the efficacy from the co-formulations.

**Table 5:** Overview STB control from mixtures and FPX and disease severity (%) in untreated (untr.) plots.

STB Control (%), mixtures and FPX, flag leaf, 2021				Untr.	Revysol	Imtrex	Questar	Revystar XL	Revytrex	Elatus ERA	Ascra Xpro EC 260	Silvron Xpro
					MFA	FXP	FPX*	FXP+MFA	FXP+MFA	BVF+PTH	BIX+FLP+PTH	BIX+FLP
Trial	Ctry.	GS	DAA	-	150 g/ha	125g/ha	100/75 g/ha	75 + 150 g/ha	100 + 100 g/ha	75 + 150 g/ha	98 + 98 + 195 g/ha	100 + 100 g/ha
21328-1	DK	77	43	70.0	65	81	80	87	80	79	79	81
21328-3	UK	75	38	8.0	100	72	100	96	100	81	96	90
21328-4	IE	75	51	91.3	90	42	95	94	92	63	88	68
21328-5	BE	75	38	86.1	82	65	71	87	92	58	75	54
21328-6	FR	75	48	92.5	87	75	85	98	98	81	90	86
21328-7	PL	75	45	55.0	75	81	72	84	91	95	92	91
21328-9	DE	75	30	29.2	91	90	84	96	97	76	87	81
21328-10	DE	83	40	10.7	65	44	73	81	71	68	78	61
STB Control (%), F -1, 2021				Untr.	MFA	FXP	FPX	FXP+MFA	FXP+MFA	BVF+PTH	BIX+FLP+PTH	BIX+FLP
21328-1	DK	75	35	47.5	89	94	94	95	94	94	94	93
21328-2	UK	69	41	30.4	79	69	86	84	89	70	82	77
21328-4	IE	65	35	63.3	69	55	66	69	58	56	60	50
21328-5	BE	75	27	45.0	91	93	96	98	96	83	91	77
21328-6	FR	71	33	24.4	93	97	96	98	99	97	99	99
21328-7	PL	71	30	68.3	89	98	81	96	97	93	93	90
21328-9	DE	69	22	36.4	90	89	91	94	93	78	89	93
21328-10	DE	70	27	5.6	61	62	59	71	59	59	66	54
Avg., flag leaf				55.4	81.7	68.5	82.4	90.4	90.0	75.2	85.6	76.5
Avg., flag leaf -1				40.1	82.2	67.0	83.7	88.2	85.7	78.7	84.1	79.0

Assessments were carried out at growth stage (GS) 65-83, 27-45 days after application (DAA) in 9 trials in 2021. Tested fungicides included mefentrifluconazole (MFA), fluxapyroxad (FXP), fenpicoxamid (FPX), fluxapyroxad + mefentrifluconazole (FXP+MFA) (Revystar XL and Revytrex), benzovindiflupyr + prothioconazole (BVF+PTH), bixafen + fluopyram + prothioconazole (BIX+FLP+PTH) and bixafen + fluopyram (BIX+FLP). Colors signify the ranking of treatment effects within individual trials. Efficacy data were ranked using a color gradient for each individual trial; the ranking should therefore be read horizontally and not vertically. Green: highest rating. Yellow: medium rating. Orange: lowest rating.

*The lower dose was used in FR and BE in accordance with the authorized rate in these countries.

*d) Effect of azoles, SDHIs, mixtures of azoles/SDHIs and fenpicoxamid on yield responses*

**Figure 6:**
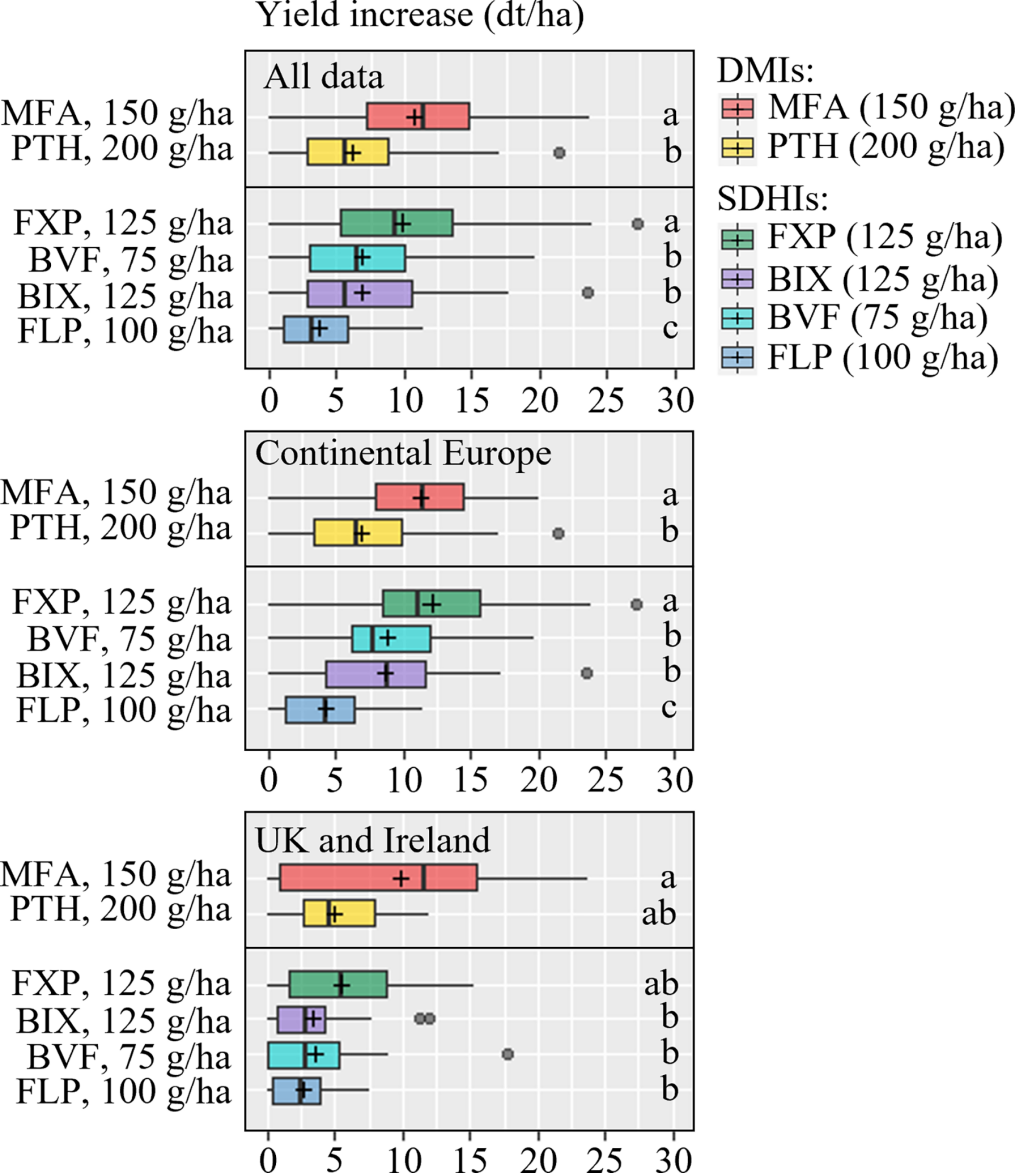
Yield increases from 21 field trials with the azoles mefentrifluconazole (MFA), prothioconazole (PTH) and the SDHIs fluxapyroxad (FXP), bixafen (BIX), fluopyram (FLP) and benzovindiflupyr (BVF). Trials from Continental Europe were carried out in Denmark (DK), France (FR), Germany (DE), Poland (PL) and Belgium (BE). FLP was not included in 2021. The line inside the boxes indicates the median and ‘+’ indicates the average. Different letters represent significant differences between treatments within boxplot as determined by Dunn’s test.

**Figure 7:**
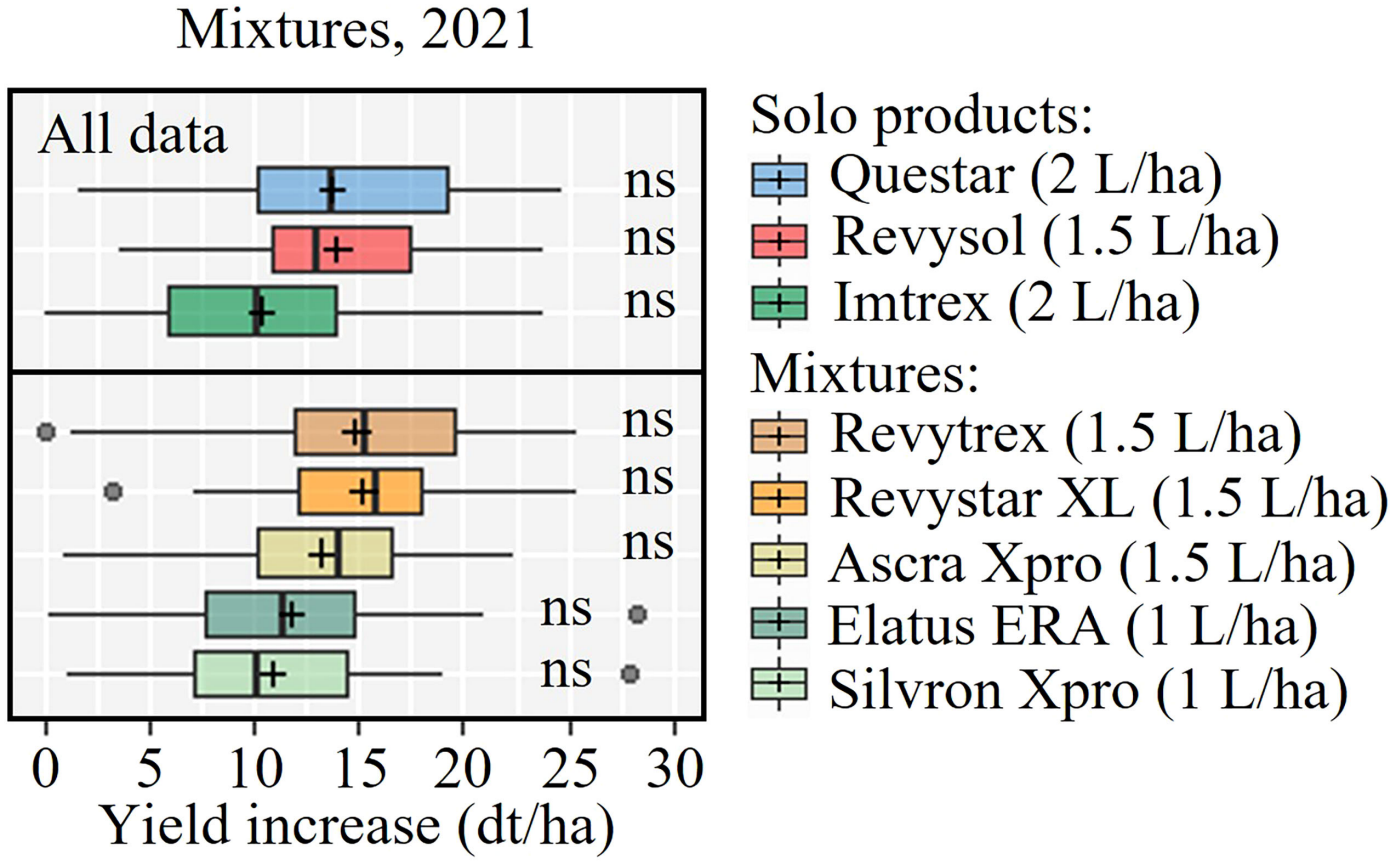
Yield increases from nine field trials with Questar (fenpicoxamid), Revysol (mefentrifluconazole), Imtrex (fluxapyroxad), Revytrex and Revystar (fluxapyroxad + mefentrifluconazole), Ascra Xpro (bixafen + fluopyram + prothioconazole), Elatus ERA (benzovindiflupyr + prothioconazole) and Silvron Xpro (bixafen + fluopyram). The line inside the boxes indicates the median and ‘+’ indicates the average. NS = no significant differences were found between treatments determined by Dunn's test.

Yields from 21 trials were included in the analysis of solo actives (**Figure 6**). Generally the yield responses were best from MFA and FXP, which overall performed better than the rest of the tested active ingredients. The data also showed that FLP was significantly inferior to BIX and BVF and PTH. The SDHIs BIX, BVF and FLP gave significantly lower yield increases than MFA in UK and Ireland, which was also the case in Continental Europe. Nevertheless, the average yield increase suggested that FXP performed more in line with MFA in Continental Europe, and FLP gave significantly lower yield increases than all other treatments. Yield responses from fungicide mixtures were tested in 9 trials in 2021 (**Figure 7**). This analysis shows that all products increased yields significantly. Still, the three solo solutions and the five mixtures gave similar increases, and no statistically significant differences were found between the tested fungicides.

### 3.3 *Zymoseptoria tritici* azole and SDHI sensitivity

#### 3.3.1 *In vitro* sensitivity

*In vitro* testing was carried out based on isolates from 29 trials. With a few exceptions ten *Z. tritici* isolates were successfully collected from each trial, and their sensitivity was analyzed (**Table S3**). These exceptions included: only four isolates were obtained from Poland in 2020 and again 2021; only five isolates were obtained from the trial in Northern Germany in 2021, only three isolates were obtained from Belgium in 2021, which was the only trial conducted in Belgium across the trial series, and given this limited number these were excluded from further analysis. The sensitivity of *Z. tritici* to MFA was only analyzed on isolates recovered in 2020 and 2021.

**Figure 8:**
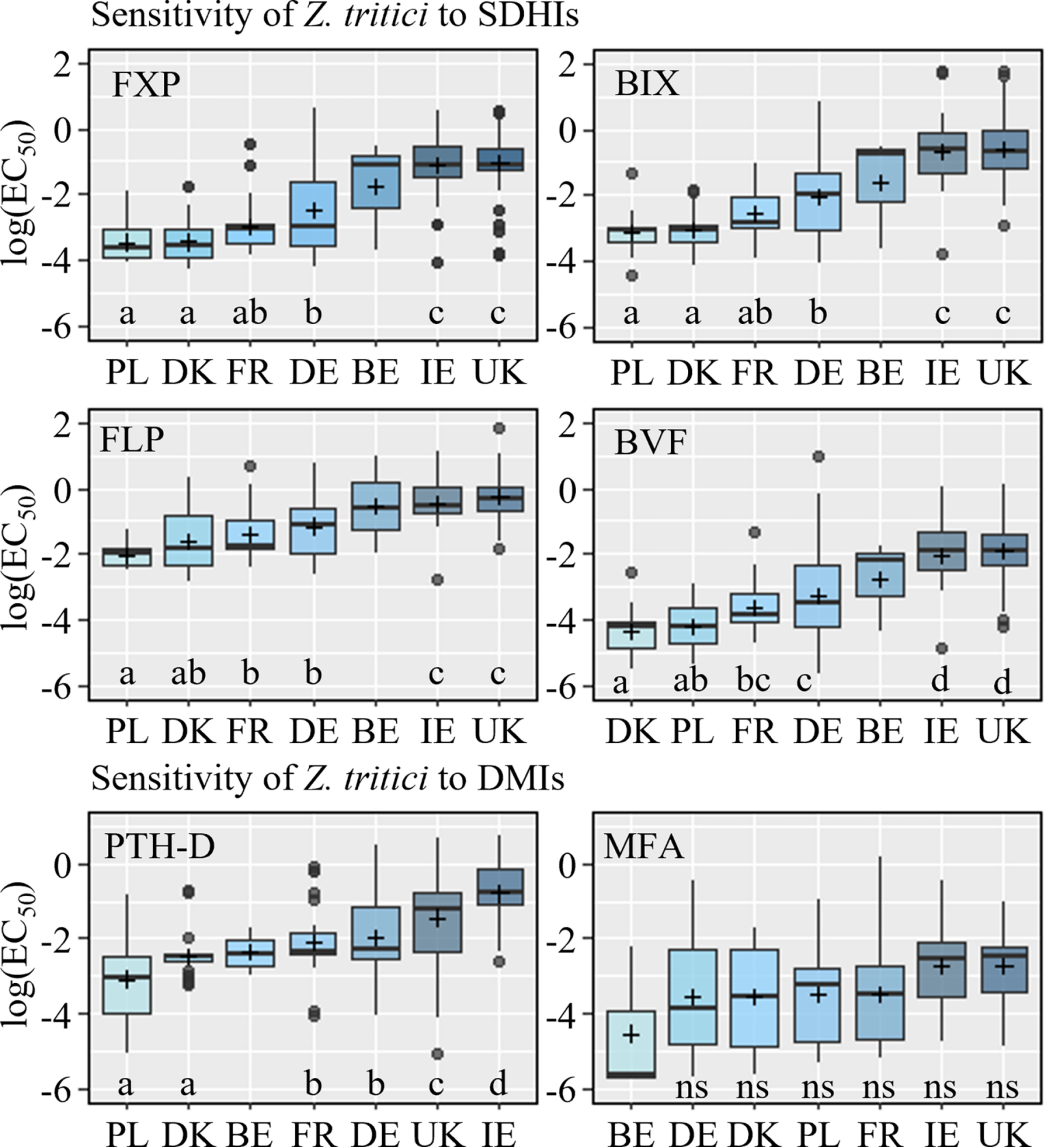
Sensitivity (log EC_50_) of *Z. tritici* isolates, collected in Europe from 2019 to 2021, to SDHI fungicides fluxapyroxad (FXP), bixafen (BIX), fluopyram (FLP), and benzovindiflupyr (BVF), and the azoles prothioconazole-desthio (PTH-D) and mefentrifluconazole (MFA). Data is divided by countries Poland (PL), Denmark (DK), France (FR), Germany (DE), Belgium (BE), Ireland (IE), and the United Kingdom (UK). Different letters below the boxes represent significant differences between treatments within the plot as determined by Dunn’s test.

Amongst the isolates wide ranges in sensitivity (EC_50_) were observed for each fungicide (BIX: 0.01 – 5.79 mg/l; BVF: 0.003 – 2.55 mg/l; FLP: 0.07 – 9.85 mg/l, PTH-D: 0.01 – 2.13 mg/l; MFA: 0.001 – 0.64 mg/l). Sensitivity also varied widely across the countries, and with the exception of MFA this variation was broadly similar for each fungicide (**Figure 8** and **Table S6**). For all compounds except MFA, the least sensitive isolates were collected from Ireland and the UK, and the most sensitive came from Denmark and Poland. This was reflected in the differences observed between the countries in terms of their sensitivity to the SDHIs. No significant differences were seen between the sensitivity of isolates from Ireland and the UK, but both countries had significantly less sensitive isolates than other countries. Statistically significant differences were identified between Denmark, France, Germany and Poland, but these were dependent on the specific SDHI (**Figure 8**), with both Denmark and Poland being the most sensitive, although not always significantly more sensitive than the collection from France. The Irish collection was significantly less sensitive to PTH-D compared to all other collections, including that from the UK. The differences between the PTH-D sensitivity of isolates from other countries were similar to those observed for the SDHIs (**Figure 8**).

No clear shift in the sensitivity was seen from 2019-2021, and no statistically significant differences were found between EC_50_ values in the different years overall. When grouped by country, significant differences were only observed among years in Germany for BVF, from which decreasing EC_50_ values were measured each year (data not shown).

**Figure 9:**
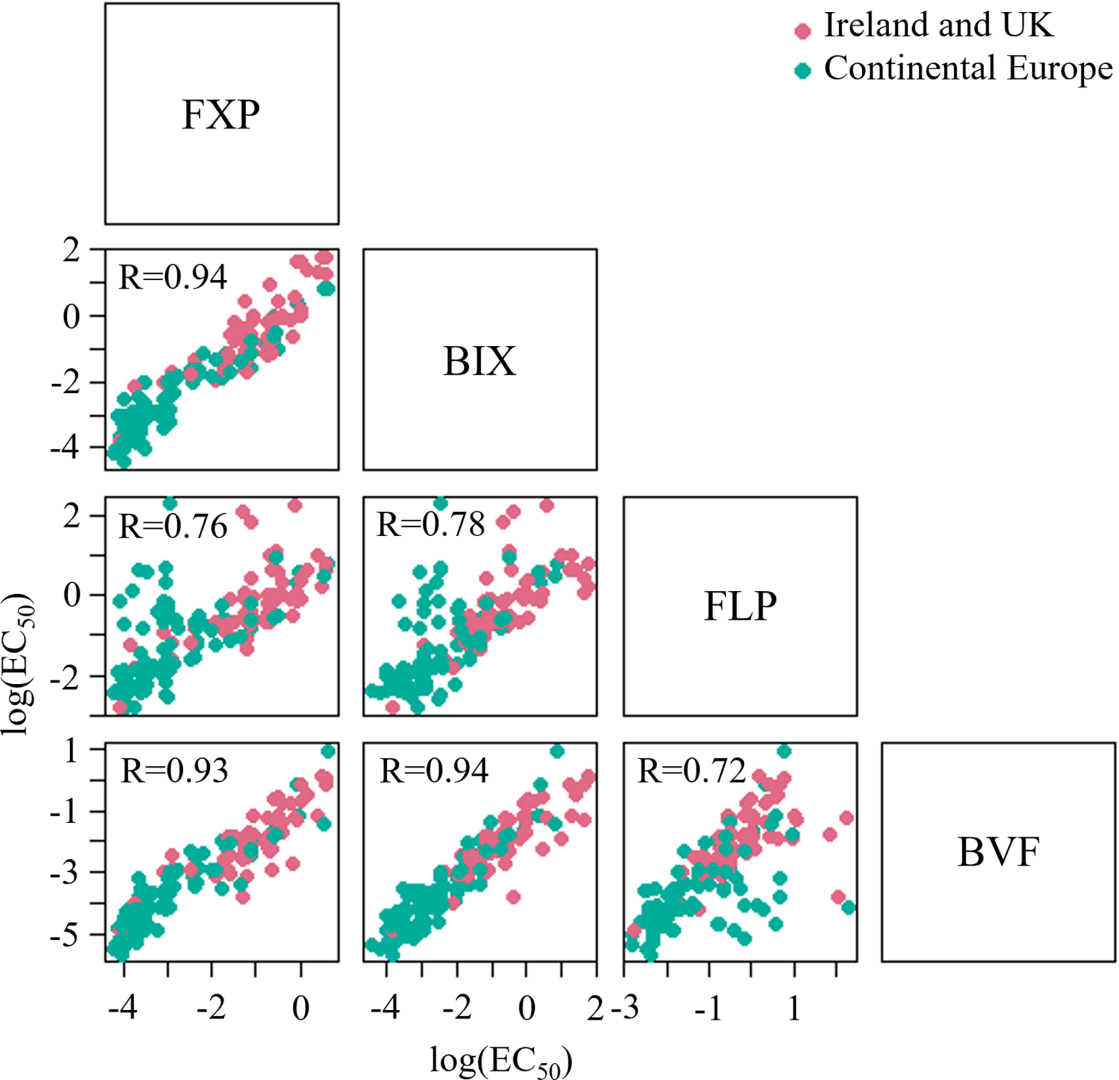
Cross-resistance between SDHI fungicides based on sensitivity data (EC_50_) from 3 seasons and 202 *Z. tritici* isolates. Samples were collected from trials located in Ireland and UK, and continental Europe, including Denmark, Germany, Belgium, France and Poland.

Very high levels of correlation were found between the sensitivity of isolates towards FXP and BIX, BIX and BVF (*r*=0.94 for both comparisons) and FXP and BVF (*r*=0.93). The correlations between FXP and FLP, BIX and FLP and FLP and BVF were slightly lower (*r* ranging from 0.72-0.76) (**Figure 9**). It was possible to analyze the correlation between MFA and PTH-D in 2020 and 2021, but no correlation was found.

Moderate levels of negative correlations were found between the average sensitivity of isolates *in vitro* (EC_50_ values) towards the specific SDHIs and the field efficacy (R^2 =^ 0.2-0.46) (data not presented).

#### 3.3.2 Target site alteration frequencies

Amongst the leaf collections analyzed no SDH-B mutations were detected, nor were the SDH-C mutations T79I and G90R. Only the SDH-C mutations T79N, W80S, N86S and H152R were found, and the frequencies varied greatly across locations (**Table 6**). It should, however, be noted that due to the limits of detection associated with the methods used only frequencies above a threshold of 5% are reported. T79N and N86S were found most frequently, while W80S and H152R were only detected in the UK, in 4 and 2 locations, respectively, with frequencies ranging from 5-15%. T79N was found only in the UK and Ireland, where this mutation was found in every trial. The frequencies of this mutation were highest in Ireland ranging from 60% to 64%, while frequencies of 13-25% were found in the UK trials. Among the SDH-C mutations, only N86S was detected in countries other than the UK and Ireland. While this mutation was found at frequencies of 11-37% in Belgium, Germany, Hungary, the Netherlands, and Poland, it was most frequently detected in the UK and Ireland. Only in one trial in 2021 in the UK was this mutation not found, and in the remaining trials of this region the frequencies ranged from 18-41%. No clear development was seen from 2019-2021.

**Table 6:** Frequencies of sdh-c and cyp51mutations.

			CYP51										SDH-C				
Year	Trial	Ctry.	D134G	V136A	V136C	A379G	I381V	S524T		Year	Trial	Ctry.	T79N	W80S	N86S	H152R
2021	21328-5	BE	53	67	21	15	95	30		2021	21328-5	BE	0	0	14	0
2019	19341-5	DE	31	38	0	21	99	32		2019	19309-7	DE	0	0	11	0
2019	19341-6	DE	53	59	10	19	98	22		2020	20334-2	DE	0	0	0	0
2020	20334-2	DE	64	74	11	17	100	24		2020	20334-3	DE	0	0	30	0
2020	20334-3	DE	11	24	28	29	100	62		2021	21328-9	DE	0	0	0	0
2021	21328-9	DE	62	71	15	22	97	24		2021	21328-10	DE	0	0	0	0
2021	21328-10	DE	25	40	17	18	100	71		2019	19309-1	DK	0	0	0	0
2019	19341-1	DK	59	68	0	12	99	5		2020	20334-1	DK	0	0	0	0
2020	20334-1	DK	40	50	13	13	99	7		2021	21328-1	DK	0	0	0	0
2021	21328-1	DK	45	54	0	10	100	5		2019	19309-6	FR	0	0	0	0
2019	19341-7	FR	42	63	0	19	79	23		2020	20334-4	FR	0	0	0	0
2020	20334-4	FR	46	61	18	11	96	24		2021	21328-6	FR	0	0	0	0
2021	21328-6	FR	42	60	18	12	95	19		2021	21328-8	HU	0	0	21	0
2021	21328-8	HU	0	0	0	38	98	0		2019	19309-5	NL	0	0	16	0
2019	19341-8	PL	14	20	0	20	96	6		2020	20334-5	PL	0	0	0	0
2020	20334-5	PL	24	35	15	11	99	23		2021	21328-7	PL	0	0	37	0
2021	21328-7	PL	26	34	13	11	100	13								
2019	19341-4	IE	45	80	0	24	97	88		2019	19309-4	IE	60	0	18	0
2020	20334-8	IE	51	93	16	46	100	97		2020	20334-8	IE	61	0	26	0
2021	21328-4	IE	62	86	15	35	100	96		2021	21328-4	IE	64	0	21	0
2019	19341-2	UK	23	42	23	36	98	63		2019	19309-2	UK	13	5	41	0
2019	19341-3	UK	33	61	19	45	98	71		2019	19309-3	UK	14	11	31	7
2020	20334-6	UK	34	60	28	42	100	52		2020	20334-6	UK	20	15	39	0
2020	20334-7	UK	36	69	35	50	98	90		2020	20334-7	UK	23	0	41	0
2021	21328-2	UK	32	58	31	52	98	66		2021	21328-2	UK	17	12	20	0
2021	21328-3	UK	38	58	34	57	100	92		2021	21328-3	UK	25	0	0	10
2019			37	54	6	24	95	39					12	3	20	1
2020			38	58	21	27	99	47					13	1.9	17	0
2021			39	53	16	27	98	42					11	1.2	11	1
Continental Europe			37	48	11	17	97	23					0	0	8	0
UK and Ireland			39	67	22	43	99	79					31	5	28	2

Screening was also carried out for the sdh-b mutations N225I, N225T, H267X, T268I, I269V, sdh-c mutations T79I and G90R which were not found at any of the locations. Colors signify the following ranges of mutation frequencies: green: 0%, light yellow: 1-20%, dark yellow: 21-40%, orange: 41-60%, red: 61-100%.

The CYP51 mutations were more evenly detected across Europe, but clear patterns were still discernible. The most widespread mutation was I381V, which was found at high frequencies in all samples. D134G and V136A were also present at high frequencies at most locations, with average frequencies of 38% and 55%, respectively, and only one Polish and one German trial had frequencies below 21%, while neither mutation was found in Hungary. A379G was found at intermediate frequencies with an average of 26% across all trials. This mutation was found at all locations, but a clear distinction was seen between the regions, and while 17% of isolates from Continental Europe carried this mutation on average, the frequency was 43% on average in The UK and Ireland. V136C was detected least frequently in an average of 15% across all trials, and the difference between the regions was less pronounced. However, in five trials located in Denmark, France, Hungary and Poland V136C was not detected, while the same was the case in one Irish trial. The greatest difference between the regions was seen for S524T, which was found with an average frequency of 23% in Continental Europe, while the average frequency was 79% in The UK and Ireland.

## 4 Discussion

Resistance emergence and gradual sensitivity shifting in *Z. tritici* populations towards SDHIs and azoles have become a widespread problem in many areas of European wheat production.This challenges current fungicide control practices, where SDHIs and azoles still are the most widely used fungicides for the control of STB (Jørgensen and Heick 2021, Turner et al., 2021). Previous studies within the Eurowheat framework have investigated both *Z. tritici* sensitivity to azoles, CYP51 mutation, and azole field efficacies, which verified that sensitivity to the azoles is variable across Europe and, in general, decreases from Eastern to Western Europe (Jørgensen et al., 2021a). A similar trend has been described by Hellin et al. (2021). The more rapid local adaptation of the *Z. tritici* populations to fungicides in countries such as Ireland and Great Britain was also confirmed in those studies, which the authors attributed to (i) a higher disease pressure and (ii) more intensive fungicide usage to combat these pressures (Hellin et al., 2021; Jørgensen and Heick, 2021b).

The present study confirms that the presence of alterations conferring resistance to azole and SDHI fungicides are widespread throughout the European *Z. tritici* populations (**Table 6**). As also observed in other studies (Rehfus et al., 2018a; Rehfus et al., 2020; FRAC, 2021; Hellin et al., 2021), the most common SDH mutations found in this study are C-T79N and C-N86S, conferring moderate resistance to *Z. tritici* (Hellin et al., 2021). The target site mutation C-H152R is causing particular interest as it drastically decreases the sensitivity to all SDHIs. In this study, C-H152R was only detected in two of the trials, both from the United Kingdom. Although previously detected in other continental European populations, some studies suggest that C-H152R comes with a fitness cost to the pathogen (Dooley et al., 2016; Rehfus et al., 2020), which makes it less likely to increase dramatically and may explain why it was not detected elsewhere in the presented trials series

The *in vitro* resistance testing in this study confirmed a reduced sensitivity towards all tested SDHIs in the Irish and British trials and that a gradual but still significant shifting also has taken place in Germany and Belgium compared with the sensitivity measured in Poland, Denmark, and France. These differences correspond to the differences in SDH-C mutations detected between the different countries. In this way, the previously seen gradient of increasing azole-resistant *Z. tritici* populations from east to west (Jørgensen et al., 2021a) can also be confirmed to apply to SDHI resistance. Previous studies have shown a good correlation between field efficacies and EC_50_ values from *in vitro* testing (Blake et al., 2018). The current study with SDHI fungicides shows a similar relationship. The current study with SDHI fungicides shows a similar relationship, however, the specific correlations between field efficacy on STB and EC_50_ values are only moderate (R^2^=0.2-0.46), indicating that also other factors like level of disease attack and timing impact the specific performances in individual trials.

Although the azole group of fungicides has been authorized for the control of foliar diseases in wheat since the late 1970s, this group is still regarded as among the most important fungicide groups available to growers. This goes for the control of diseases in wheat but also for most other major crops (Jørgensen and Heick, 2021b). Despite the erosion of efficacy measured for most azoles, it is also clear that the levels of efficacy they bring differ depending on the CYP51 mutations dominating the different European populations. Data collected since 2015 have confirmed that when applied at full rates, the older generation of azoles (epoxiconazole, metconazole, tebuconazole, difenoconazole, and prothioconazole) still provide moderate control (typically 40-70%) of STB (Jørgensen et al., 2018; Jørgensen et al., 2020). However, it is also clear that their field efficacy has been reduced (Kildea, 2016; Heick et al., 2017; Blake et al., 2018).

Prothioconazole is the second newest azole on the European fungicide market and has been used intensively as part of cereal disease control strategies for approximately 20 years (Jørgensen and Heick, 2021). In the current study, PTH still provided moderate control with an average of ca. 45% control from a single application at GS 37-39, although with a wide range of control observed (11-84%), depending on disease pressure, site, and season. The newest azole on the market, MFA, was authorized for control of STB in 2021. In the presented trials it provided good control of STB with an average of 82% control, covering a range of 41-100%. These results align with previous trials, where MFA similarly outperformed the older azoles giving an average control of ca. 80% (Jørgensen et al., 2020). As shown in this study, the reduced efficacy from PTH not only impacted field control of STB, but also gave reduced yields compared to the more effective azole, MFA. On average, PTH yielded 107.2% (5.9 dt/ha) and MFA 112.3% (10.3 dt/ha). These differences are more pronounced than the differences seen by Jørgensen et al. (2020), where MFA only yielded 3 dt/ha more than PTH based on 17 trials across Europe in 2017-18. Despite a difference in cost between products with PTH and MFA, it is still clear that MFA will be a more profitable treatment than PTH.

Mefentrifluconazole has been seen to have a stronger binding efficiency ensuring good control even of highly shifted isolates with complex *CYP51-*haplotypes (Strobel et al., 2020). It is currently unclear whether MFA selects for specific alterations. Still, cross-resistance studies have shown a high correlation between MFA and difenoconazole/tebuconazole (Heick et al., 2020), and the effects of these actives are strongly impeded by alterations such as D134G and V136C.

As a result of historical experiences and the previously provided anti-resistance strategies, it is still strongly recommended to include anti-resistance strategies for MFA. These should include elements of not using the product repeatedly during the season- preferably once per season, avoiding applying solo products, using mixtures where all mixing partners are equally effective against STB, and not using it at excessive rates (van den Bosch et al., 2014).

### 4.1 Dose effect

An overall dose effect was observed for all tested fungicides in the five trials from 2019, where full and half rates were compared (**Figure 4** and **Table S5**), although differences were not significant as shown in **Figure 4**. The data showed a clearer dose-response for some active ingredients than for others, which also indicates that specific actives have a more considerable reduction potential than others. Specifically, for PTH, FLP, and BVF the impact from dose rates was relatively minor, around 10% or less, while it was more pronounced for the more potent products like MFA, FXP, and BIX. It is, however, worth mentioning that despite always giving inferior control on average, reducing the dose by 50% did not significantly impact the efficacy of any of the tested actives. Overall, a slightly poorer control was seen on the F-1 compared to the flag leaves, which given the treatment timing at GS 37-39, represented a more curative control on F-1, and a more preventive control on the flag leaves, which is in accordance with other investigations from the UK and Ireland (Blake et al., 2018).

Overall, keeping down the dose might be desirable to reduce environmental impact and selection for target site selection in accordance with van den Bosch et al. (2014). The economically optimal rate for any given fungicide type is highly variable and depends on grain price, site, seasons, and cultivars. Results from Ireland suggest that varieties exhibiting strong STB resistance may allow confident reductions in fungicide programs (Lynch et al., 2017).The use of fungicides at less than the recommended dose has been widely adopted in many countries (Jørgensen et al., 2017). The basis on which those dose decisions are being made is less clear, suggesting a gap between research and its translation into practice. The presented data in this paper show that the potential for reductions is variable and more likely for effective mixtures than for solo solutions giving low to moderate control as presented in this paper. Practical farming in Western Europe uses typically 2-3 treatments per season. These treatments will consist of various products, most likely co-formulations, which include all available modes of action to ensure sufficient disease control.

### 4.2 Co-formulations

The tested co-formulations of an SDHI with an azole and showed more stable and improved control compared with either solo azoles or SDHIs (**Figure 5** and **Table 5**). This is in line with previous findings from Ireland, which have shown that averaged across the margin above fungicide cost was greater for crops treated with the azole + SDHI treatment than an azole-only treatment for all varieties evaluated (Lynch et al., 2017).

If looking specifically at the UK and Irish sites and one of the German trials, which had EC_50_ values for both chemistries, the efficacy from solo SDHIs was reduced (**Figure 3**). This likely confirms a link between reduced efficacy and higher EC_50_ values. It is therefore recommended to apply co-formulations, as these will provide a more stable level of disease control and are expected to slow down resistance development, as described by van den Bosch et al. (2014). In regions now already challenged with shifs in sensitivity to both SDHIs and azoles, co-formulations can help maintain disease control, whilst in regions not yet challenged they will delay such shifting from occurring.

The benefits from co-formulations can, apart from the actives giving a broader control, also be the result of increased dose input. The SDHI co-formulation Silvron Xpro, including the two SDHIs BIX and FLP was also included in the trials to compare the efficacy of the SDHI component of the three-way mixture product, Ascra Xpro which also contains the azole PTH and is widely applied throughout Europe. The co-formulation of BIX and FLP performed well and better than any of the solo SDHIs. Adding PTH to the two SDHIs increased control only slightly by 5-8%. Yield data from the co-formulation trials showed a similar pattern of treatment responses as seen for the field effects on STB. The yield increases were on average between 1 and 1.5 T/ha. The variation of yield increases from the three solo products and the five mixtures was limited, and no statistically significant differences were found between them (**Figure 7**). Treatment cost varies across Europe depending on specific national conditions, grain prices, etc. However, with a roughly estimated treatment cost equivalent to 2-4 dt/ha, the solo treatments applied at GS 39 in this project have generally been very profitable and cost-effective.

The QiI fungicide FPX was included and compared with the efficacy of the different co-formulations. Although FPX performed in line with the better co-formulations, it is also important to emphasize that this active ingredient should not be used as a solo treatment. Resistance risk evaluations of FPX have shown that the product also is at risk of developing resistance and therefore only should be used in mixtures with other actives and only once per season (Owen et al., 2017; Fouché et al., 2022).

### 4.3 Cross-resistance

The degree of cross-resistance among SDHIs for several fungal pathogens has been discussed intensively (Yamashita and Fraaije, 2018). The current study confirms a clear cross-resistance between all tested SDHIs. Although significant positive cross-resistance was identified between FLP and BIX/FXP the scatterplots of these relationships indicated the presence of outliers that did not follow the overall positive pattern. Other studies have found FLP not always showing a clear cross-resistance pattern with other SDHIs (Yamashita and Fraaije, 2018; Steinhauer et al., 2019). It is generally accepted that cross-resistance patterns among SDHIs are complex and will continue to be so as many mutations confer full cross-resistance while others do not. The nature of the mutations found in populations varies with species and the selection compound used, but cross-resistance between all SDHIs has to be assumed at the population level (Sierotzki and Scalliet, 2013). Specifically in relation to FLP, a pre-existing non-target site resistance mechanism has been identified in *Z. tritici*, which has been found to be present at variable levels in different field populations (Yamashita and Fraaije, 2018), this might have impacted the lower levels of cross-resistance also seen in this study.

## 5 Conclusion

European testing of four SDHI fungicides for control of STB during three seasons confirms that shifting in fungicide efficacy against STB is challenged in some countries, while the efficacy is still good in other countries. The efficacy assessments based on field data, supported by *in vitro* testing, also clearly confirmed a significant shifting in *Z. tritici’s* sensitivity from Eastern to Western Europe for all SDHIs tested. These changes were supported by differences in the composition of specific SDH-C mutations between local *Z. tritici* populations. A high degree of cross-resistance between the SDHIs tested was seen based on EC_50_ values; however, the values of FLP were slightly less correlated with those of the other actives. Across all European countries, the azole MFA performed better for control of STB than PTH. The sensitivity to PTH also showed an East-West gradient across Europe based both on EC_50_ values and CYP51 mutations. The sensitivity to MFA did not differ significantly between the included countries. The reduced efficacy from both PTH and the tested SDHIs also impacted the yield responses, which were substantially reduced in Ireland and the UK compared with continental Europe. Co-formulations based on azoles and SDHIs, and the new QiI fungicide FPX proved to be the most effective solutions for the control of STB. This benefit was clearest where the populations had shifted to lower sensitivity to both azoles and SDHIs.

## Data availability statement

The raw data supporting the conclusions of this article will be made available by the authors, without undue reservation.

## Author contributions

LJ and NM organized the study and ensured the trials were carried out following common protocols. LJ, NM, AO’D, KW, JB, MG, CM, RB, SW, SK, CB, PH, BR and GC, were responsible for the trials carried out by specific partners. NM and LJ analyzed the data. LJ, NM, TH, SK, and PH drafted the manuscript. All authors contributed to the article and approved the submitted version.

## Funding

The project has been financed by BASF SE. The project organization and steering were led by Aarhus University and involved partners were contracted by Aarhus University.

## Acknowledgments

The authors would like to thank all the technicians involved in the trial work, and BASF SE, who financed the project and for collaboration with Dr. Rosie Bryson and Mr. Dieter Strobel from BASF.

## Conflict of interest

GS was employed by company BASF. JB was employed by company ADAS.

The remaining authors declare that the research was conducted in the absence of any commercial or financial relationships that could be construed as a potential conflict of interest.

Although BASF funded this project, their primary role has been to assist in analysing specific target site alterations and provide the tested chemicals. The products included in the testing represent key fungicide actives and co-formulations belonging to both BASF and their competitors. BASF did not influence the experimental design, selection of locations, or computation and presentation of data.

## Publisher’s note

All claims expressed in this article are solely those of the authors and do not necessarily represent those of their affiliated organizations, or those of the publisher, the editors and the reviewers. Any product that may be evaluated in this article, or claim that may be made by its manufacturer, is not guaranteed or endorsed by the publisher.

## Abbreviations

BIX, bixafen; BVF, benzovindiflupyr; FLP, fluopyram; FPX, fenpicoxamid; FXP, fluxapyroxad; MFA, mefentrifluconazole; PTH, prothioconazole; PTH-D, prothioconazole-desthio; BE, Belgium; DE, Germany; DK, Denmark; FR, France; HU, Hungary; IE, Ireland; NL, the Netherlands; PL, Poland; UK, The United Kingdom.
