# Supplementary material for: Shifting sensitivity of septoria tritici blotch compromises field performance and yield of main fungicides in Europe
Source: Front Plant Sci. 2022 Nov 22;13:1060428. doi: 10.3389/fpls.2022.1060428 (PMC9723467; doi:10.3389/fpls.2022.1060428)
Supplement: Supplementary file 1 [file DataSheet_1.docx]

# Supplementary Material

**Table S1.** Background information for all 33 trials. The information includes location, cultivars, spraying/sowing/harvest date, previous crop. Trials included in individual datasets are indicated by ‘+’ and ‘-’, which indicates whether data have been collected regarding disease severity on flag leaves (STB, F), on flag leaves -1 (STB, F-1), fungicide sensitivity (EC_50_), SDH-C/B and/or CYP51 mutation frequencies (Mutation). *Only molecular data were considered.

| Year | Trial | Ctry. | Institution | Location | Cultivar | Spraying date | Sowing date | Harvest date | Precrop | STB, F | STB, F-1 | EC_50_ | Mutation |
| --- | --- | --- | --- | --- | --- | --- | --- | --- | --- | --- | --- | --- | --- |
| 2019 | 19309-1 | DK | AU | Flakkebjerg | Hereford | 23.05.19 | 13.09.18 | 04.08.19 | HORVS | + | + | + | + |
|  | 19309-2 | UK | NIAB | Sutton Scotney | Elation | 20.05.19 | 21.10.18 | 23.08.19 | BRSNW | - | - | - | + |
|  | 19309-3 | UK | ADAS | Bodenham | Santiago | 05.06.19 | 02.10.19 | 27.08.19 | BRSNW | + | + | + | + |
|  | 19309-4 | IE | Teagasc | Oak Park | JB Diego | 28.05.18 | 26.10.17 | 15.08.18 | PHSVX | + | - | + | + |
|  | 19309-5 | NL | Wageningen | Lelystad | Expert | 15.05.19 | 25.10.18 | 23.07.19 | TRZAW | - | - | - | + |
|  | 19309-6 | FR | Arvalis | Aubigny-Aux-Kaisnes | Sacramento | 03.05.18 | 16.10.19 | 22.07.19 | SOLTU | + | + | + | + |
|  | 19309-7 | DE | EAS | Pölitz | Tobak | 23.05.19 | 28.09.18 | 13.08.19 | TRZAW | + | + | + | + |
|  | 19341-1 | DK | AU | Flakkebjerg | Kalmar | 14.05.19 | 24.09.17 | 03.08.18 | BRSNW | - | - | +* | +* |
|  | 19341-2 | UK | NIAB | Aby | KWS Santiago | 24.05.19 | 26.09.18 | 20.08.19 | BRSNW | - | - | +* | +* |
|  | 19341-3 | UK | ADAS | Bishop’s Froome | Santiago | 13.05.19 | 10.10.18 | 27.08.19 | BRSNW | - | - | +* | +* |
|  | 19341-4 | IE | Teagasc | Meath | JB Diego | 28.05.18 | 26.10.17 | 15.08.18 | PHSVX | - | - | +* | +* |
|  | 19341-5 | DE | JKI | Sickte | Bergamo | 24.05.19 | 17.10.18 | 24.07.19 | BRSNN | - | - | - | +* |
|  | 19341-6 | DE | LfL | Fraunberg | Kometus | 24.05.19 | 10.10.18 | 25.07.19 | ZEAMX | - | - | +* | +* |
|  | 19341-7 | FR | EAS | Val-en-Viges | Descartes | 03.05.19 | 25.10.18 | 11.07.19 | SORVU | - | - | +* | +* |
|  | 19341-8 | PL | Sosnicowice | Łany Wielkie | Fidelius | 17.05.19 | 28.09.18 | 17.07.19 | BRSNW | - | - | +* | +* |
| 2020 | 20334-1 | DK | AU | Flakkebjerg | Hereford | 26.05.19 | 22.09.19 | 12.08.20 | HORVS | + | + | + | + |
|  | 20334-2 | DE | LfL | Frankendorf | Kometus | 18.05.20 | 15.10.19 | 07.08.20 | BRSNW | - | + | + | + |
|  | 20334-3 | DE | EAS | Rethwischfeld | JB Asano | 20.05.20 | 20.09.19 | 08.08.20 | BRSNW | + | + | + | + |
|  | 20334-4 | FR | Arvalis | Dury | RGT Sacramento | 27.04.20 | 30.10.19 | 17.07.20 | SOLTU | + | + | + | + |
|  | 20334-5 | PL | Sosnicowice | Łany Wielkie | Fidelius | 08.05.20 | 24.09.19 | 28.07.20 | BRSNW | + | + | + | + |
|  | 20334-6 | UK | ADAS | Ledicot | Gravity | 17.05.20 | 10.10.19 | 11.08.20 | AVESA | - | + | + | + |
|  | 20334-7 | UK | NIAB | Sutton Scotney | Gravity | 12.05.20 | 23.10.19 | 05.08.20 | BRSNW | - | - | + | + |
|  | 20334-8 | IE | Teagasc | Carlow | Costello | 27.05.20 | 14.11.20 | 13.08.20 | PHSVX | + | + | + | + |
| 2021 | 21328-1 | DK | AU | Flakkebjerg | Hereford | 27.05.19 | 14.09.20 | 21.08.21 | BRSNW | + | + | + | + |
|  | 21328-2 | UK | ADAS | Rosemaund | Gravity | 14.05.21 | 25.09.20 | 25.08.21 | BRSNW | - | + | + | + |
|  | 21328-3 | UK | NIAB | Sutton Scotney | Elation | 27.05.21 | 14.10.20 | 01.09.21 | BRSNW | + | - | + | + |
|  | 21328-4 | IE | Teagasc | Carlow | Costello | 25.05.21 | 22.10.20 | 17.08.21 | PHSVX | + | + | + | + |
|  | 21328-5 | BE | CRA-W | Clermont | Bergamo | 01.06.21 | 15.11.20 | 13.08.21 | BEAVA | + | + | + | + |
|  | 21328-6 | FR | Arvalis | Aubigny-aux-Kaisnes | RGT Sacramento | 12.05.21 | 19.10.20 | 23.07.21 | SOLTU | + | + | + | + |
|  | 21328-7 | PL | Sosnicowice | Łany Wielkie | Fidelius | 21.05.21 | 23.09.20 | 10.08.21 | BRSNW | + | + | + | + |
|  | 21328-8 | HU | MATE | Szeged | GK Arató | 10.05.21 | 10.10.20 | 06.07.21 | BRSNW | - | - | - | + |
|  | 21328-9 | DE | LfL | Fraunberg | Kometus | 31.05.21 | 09.10.20 | 10.08.21 | ZEAMX | + | + | + | + |
|  | 21328-10 | DE | JKI | Wolfenbüttel | JB Asano | 21.05.21 | 08.10.20 | 02.08.21 | BRSNW | + | + | + | + |

**Table S2.** Number of trials with usable data on STB control on flag leaves and flag -1 leaves (STB, F and STB, F-1), EC_50_ and mutation frequencies sorted by year and region.

|  | STB, F | STB, F-1 | EC_50_ | Mutation |
| --- | --- | --- | --- | --- |
| 2019 | 5 | 4 | 12 | 15 |
| 2020 | 5 | 7 | 8 | 8 |
| 2021 | 8 | 8 | 9 | 10 |
| Ireland and UK | 6 | 7 | 13 | 14 |
| Continental Europe | 12 | 12 | 16 | 19 |
| All | 18 | 19 | 29 | 33 |

**Table S3.** Number of STB isolates used for measuring EC_50_ values.

| Ctry. | FXP | BIX | FLP | BVF | PTH_D | MFA |
| --- | --- | --- | --- | --- | --- | --- |
| PL | 14 | 14 | 14 | 14 | 24 | 14 |
| DK | 30 | 30 | 30 | 30 | 30 | 20 |
| FR | 30 | 30 | 30 | 30 | 30 | 20 |
| DE | 45 | 45 | 45 | 45 | 45 | 35 |
| BE | 3 | 3 | 3 | 3 | 3 | 3 |
| IE | 30 | 30 | 30 | 30 | 30 | 20 |
| UK | 50 | 50 | 50 | 50 | 60 | 40 |

**Table S4.** Disease severity in untreated plots on flag leaves (F), flag leaves -1 (F-1), at growth stage (GS) 63-85 and 22-58 days after application (DAA).

| Year | Trial | Country | STB, F | GS | DAA | STB, F-1 | GS | DAA |
| --- | --- | --- | --- | --- | --- | --- | --- | --- |
| 2019 | 19309-1 | DK | 95.0 | 75 | 43 | 86.3 | 73 | 33 |
|  | 19309-2 | UK | 0.8 | 79 | 50 | 1.8 | 79 | 50 |
|  | 19309-3 | UK | 4.1 | 75 | 38 | 61.1 | 75 | 40 |
|  | 19309-4 | IE | 58.3 | 75 | 28 | - | - | - |
|  | 19309-5 | NL | 0.25 | 63 | 30 | 0.4 | 63 | 30 |
|  | 19309-6 | FR | 65.9 | 75 | 54 | 92.5 | 75 | 54 |
|  | 19309-7 | DE | 7.6 | 73 | 30 | 77.1 | 73 | 30 |
| 2020 | 20334-1 | DK | 61.3 | 75 | 42 | 85 | 75 | 42 |
|  | 20334-2 | DE | 1.7 | 69 | 28 | 27.3 | 75 | 37 |
|  | 20334-3 | DE | 17.5 | 75-77 | 43 | 17.5 | 75-77 | 43 |
|  | 20334-4 | FR | 6.2 | 85 | 58 | 6.3 | 75 | 44 |
|  | 20334-5 | PL | 31.3 | 75 | 48 | 40.5 | 75 | 48 |
|  | 20334-6 | UK | 0.3 | 78 | 43 | 5.7 | 78 | 43 |
|  | 20334-7 | UK | 0 | - | - | 0.8 | 69 | 34 |
|  | 20334-8 | IE | 18.6 | 75 | 49 | 32.3 | 75 | 49 |
| 2021 | 21328-1 | DK | 70 | 77 | 43 | 47.5 | 75 | 35 |
|  | 21328-2 | UK | 1.8 | 69 | 41 | 30.4 | 69 | 41 |
|  | 21328-3 | UK | 8 | 75 | 38 | 10.8 | 65 | 27 |
|  | 21328-4 | IE | 91.3 | 75 | 51 | 63.3 | 75 | 51 |
|  | 21328-5 | BE | 86.1 | 85 | 38 | 45 | 75 | 27 |
|  | 21328-6 | FR | 92.4 | 75 | 48 | 24.4 | 71 | 33 |
|  | 21328-7 | PL | 55 | 75 | 45 | 68.3 | 71 | 30 |
|  | 21328-8 | HU | 0.1 | 75-77 | 35 | 0.8 | 75-77 | 35 |
|  | 21328-9 | DE | 29.2 | 75 | 30 | 36.4 | 69 | 22 |
|  | 21328-10 | DE | 10.7 | 83 | 40 | 5.6 | 70 | 27 |
|  |  |  |  |  |  |  |  |  |

**Table S5**. Overview STB control from reduced doses and disease severity (%) in untreated (untr.) plots. Flag leaves (F, top) and flag leaves minus one (F-1, bottom) were assessed at growth stage (GS) 73-75, 30-55 days after application (DAA) in 5 trials in 2019. Tested fungicides included fluxapyroxad (FXP), bixafen (BIX), benzovindiflupyr (BVF), fluopyram (FLP), mefentrifluconazole (MFA) and prothioconazole (PTH). Colors signify ranking of treatment effects within individual trials. Efficacy data was ranked using a color gradient for each individual trial; the ranking should therefore be read horizontally and not vertically. Green: highest rating. Yellow: medium rating. Orange: lowest rating.

| STB Control (%), F | | | | Untr. | | FXP | | BIX | | BVF | | FLP | | PTH | | MFA | |
| --- | --- | --- | --- | --- | --- | --- | --- | --- | --- | --- | --- | --- | --- | --- | --- | --- | --- |
| Trial | Ctry. | GS | DAA | |  | 62.5 g/ha | 125 g/ha | 62.5 g/ha | 125 g/ha | 37,5 g/ha | 75 g/ha | 50 g/ha | 100 g/ha | 100 g/ha | 200 g/ha | 75 g/ha | 150 g/ha |
| 19309-1 | DK | 75 | 43 | | 95 | 72 | 88 | 37 | 68 | 8 | 17 | 3 | 9 | 4 | 11 | 68 | 84 |
| 19309-3 | UK | 75 | 38 | | 4.1 | 18 | 36 | 1 | 13 | 18 | 17 | 9 | 13 | 12 | 13 | 42 | 41 |
| 19309-4 | IE | 75 | 28 | | 58.3 | 62 | 39 | 40 | 47 | 37 | 38 | 33 | 15 | 43 | 25 | 61 | 76 |
| 19309-6 | FR | 75 | 55 | | 65.9 | 91 | 97 | 60 | 70 | 61 | 86 | 4 | 34 | 11 | 34 | 87 | 94 |
| 19309-7 | DE | 73 | 30 | | 7.7 | 73 | 100 | 52 | 69 | 47 | 61 | 25 | 26 | 49 | 70 | 64 | 79 |
| STB Control (%), F -1 | | | | Untr. | | FXP |  | BIX |  | BVF |  | FLP |  | PTH |  | MFA |  |
| 19309-1 | DK | 73 | 33 | | 86.3 | 76 | 86 | 31 | 62 | 33 | 42 | 6 | 20 | 13 | 20 | 71 | 83 |
| 19309-3 | UK | 75 | 38 | | 61.1 | 20 | 23 | 9 | 12 | 1 | 8 | 10 | 11 | 3 | 11 | 32 | 50 |
| 19309-6 | FR | 75 | 55 | | 92.5 | 59 | 83 | 31 | 43 | 26 | 56 | 3 | 8 | 9 | 13 | 47 | 72 |
| 19309-7 | DE | 73 | 30 | | 77.1 | 28 | 90 | 16 | 28 | 19 | 19 | 13 | 12 | 17 | 30 | 27 | 59 |
| Avg. Control (%), F | | | | 45.2 | | 61.9 | 70.7 | 37.0 | 52.4 | 32.9 | 41.6 | 15.2 | 18.9 | 24.4 | 30.2 | 63.2 | 73.9 |
| Avg. Control (%), F-1 | | | | 78.4 | | 45.1 | 69.6 | 21.2 | 35.9 | 19.4 | 29.5 | 8.2 | 13.3 | 10.6 | 18.9 | 44.0 | 65.6 |

**Table S6**. Sensitivity of STB isolates to SDHI fungicides fluxapyroxad (FXP), bixafen (BIX), fluopyram (FLP) and benzovindiflupyr (BVF), and the azoles sprothioconazole-desthio (PTH-D) and mefentrifluconazole (MFA) across Europe. Colors signify the following ranges of EC_50_ values for SDHs: Dark green: <0.09, light green: 0.10-0.29, yellow: 0.30-0.99, orange: 1.00-3.00, red: >3. For the azoles: Dark green: <0.03, light green: 0.03-0.09, yellow: 0.10-0.29, orange: 0.30-1.00, red: >1.00.

| Year | Trial | Ctry. | FXP | min-max | BIX | min-max | FLP | min-max | BVF | min-max |  | Year | Trial | Ctry. | PTH_D | min-max | MFA | min-max |
| --- | --- | --- | --- | --- | --- | --- | --- | --- | --- | --- | --- | --- | --- | --- | --- | --- | --- | --- |
| 2021 | 21328-5 | BE | 0.31 | 0.03-0.57 | 0.37 | 0.03-0.59 | 1.11 | 0.14-2.64 | 0.10 | 0.01-0.17 |  | 2021 | 21328-5 | BE | 0.11 | 0.05-0.18 | 0.04 | 0-0.11 |
| 2019 | 19309-7 | DE | 0.37 | 0.05-1.9 | 0.54 | 0.09-2.31 | 0.70 | 0.21-2.18 | 0.40 | 0.02-2.55 |  | 2019 | 19341-6 | DE | 0.36 | 0.02-1.6 | - | - |
| 2020 | 20334-2 | DE | 0.07 | 0.02-0.31 | 0.11 | 0.02-0.29 | 0.44 | 0.07-2.01 | 0.03 | 0-0.09 |  | 2020 | 20334-2 | DE | 0.19 | 0.06-0.44 | 0.02 | 0-0.06 |
| 2020 | 20334-3 | DE | 0.29 | 0.02-0.99 | 0.39 | 0.02-1.35 | 0.58 | 0.09-1.77 | 0.11 | 0-0.3 |  | 2020 | 20334-3 | DE | 0.24 | 0.02-0.48 | 0.16 | 0-0.64 |
| 2021 | 21328-9 | DE | 0.21 | 0.02-1.69 | 0.28 | 0.02-2.3 | 0.46 | 0.1-1.78 | 0.05 | 0.01-0.23 |  | 2021 | 21328-9 | DE | 0.31 | 0.04-1.65 | 0.05 | 0-0.26 |
| 2021 | 21328-10 | DE | 0.18 | 0.03-0.54 | 0.19 | 0.04-0.55 | 0.25 | 0.09-0.56 | 0.05 | 0.01-0.16 |  | 2021 | 21328-10 | DE | 0.07 | 0.02-0.18 | 0.14 | 0.01-0.33 |
| 2019 | 19309-1 | DK | 0.03 | 0.01-0.05 | 0.05 | 0.02-0.13 | 0.23 | 0.08-0.65 | 0.02 | 0-0.03 |  | 2019 | 19341-1 | DK | 0.08 | 0.07-0.1 | - | - |
| 2020 | 20334-1 | DK | 0.03 | 0.02-0.1 | 0.06 | 0.02-0.17 | 1.29 | 0.1-9.85 | 0.02 | 0.01-0.08 |  | 2020 | 20334-1 | DK | 0.16 | 0.06-0.49 | 0.05 | 0.01-0.18 |
| 2021 | 21328-1 | DK | 0.05 | 0.02-0.17 | 0.06 | 0.02-0.15 | 0.33 | 0.06-1.36 | 0.01 | 0-0.03 |  | 2021 | 21328-1 | DK | 0.07 | 0.04-0.14 | 0.07 | 0-0.17 |
| 2019 | 19309-6 | FR | 0.05 | 0.03-0.09 | 0.08 | 0.03-0.16 | 0.20 | 0.09-0.43 | 0.03 | 0.02-0.05 |  | 2019 | 19341-7 | FR | 0.11 | 0.08-0.19 | - | - |
| 2020 | 20334-4 | FR | 0.10 | 0.02-0.61 | 0.12 | 0.05-0.36 | 0.39 | 0.1-1.15 | 0.04 | 0.01-0.26 |  | 2020 | 20334-4 | FR | 0.20 | 0.02-0.8 | 0.06 | 0.01-0.16 |
| 2021 | 21328-6 | FR | 0.08 | 0.03-0.33 | 0.10 | 0.02-0.32 | 0.47 | 0.1-1.94 | 0.04 | 0.01-0.1 |  | 2021 | 21328-6 | FR | 0.28 | 0.02-0.89 | 0.15 | 0.01-1.15 |
| 2020 | 20334-5 | PL | 0.03 | 0.02-0.05 | 0.06 | 0.04-0.09 | 0.13 | 0.08-0.17 | 0.02 | 0.01-0.03 |  | 2019 | 19341-8 | PL | 0.04 | 0.01-0.13 | - | - |
| 2021 | 21328-7 | PL | 0.04 | 0.02-0.15 | 0.06 | 0.01-0.26 | 0.14 | 0.09-0.3 | 0.02 | 0-0.05 |  | 2020 | 20334-5 | PL | 0.14 | 0.04-0.43 | 0.05 | 0.02-0.06 |
|  |  |  |  |  |  |  |  |  |  |  |  | 2021 | 21328-7 | PL | 0.08 | 0.02-0.18 | 0.08 | 0.01-0.37 |
| 2019 | 19309-4 | IE | 0.32 | 0.05-0.61 | 0.58 | 0.15-1.57 | 0.63 | 0.3-1.8 | 0.21 | 0.06-0.55 |  | 2019 | 19341-4 | IE | 0.56 | 0.08-1.59 | - | - |
| 2020 | 20334-8 | IE | 0.60 | 0.2-1.76 | 1.63 | 0.27-5.78 | 1.20 | 0.36-2.97 | 0.26 | 0.05-1.03 |  | 2020 | 20334-8 | IE | 0.51 | 0.09-1.72 | 0.10 | 0.01-0.29 |
| 2021 | 21328-4 | IE | 0.40 | 0.02-0.84 | 0.44 | 0.02-0.98 | 0.58 | 0.06-1.03 | 0.11 | 0.01-0.31 |  | 2021 | 21328-4 | IE | 0.84 | 0.15-2.13 | 0.13 | 0.02-0.64 |
| 2019 | 19309-3 | UK | 0.55 | 0.02-1.77 | 0.81 | 0.05-3.49 | 0.79 | 0.2-1.93 | 0.27 | 0.01-0.87 |  | 2019 | 19341-2 | UK | 0.27 | 0.09-0.89 | - | - |
| 2020 | 20334-6 | UK | 0.39 | 0.08-1.14 | 0.79 | 0.14-3.95 | 0.78 | 0.31-1.85 | 0.16 | 0.04-0.6 |  | 2019 | 19341-3 | UK | 0.51 | 0.09-1.84 | - | - |
| 2020 | 20334-7 | UK | 0.36 | 0.02-1.02 | 0.85 | 0.12-5.15 | 0.74 | 0.17-1.55 | 0.18 | 0.02-0.85 |  | 2020 | 20334-6 | UK | 0.52 | 0.08-1.4 | 0.09 | 0.03-0.35 |
| 2021 | 21328-2 | UK | 0.69 | 0.27-1.63 | 1.84 | 0.31-5.79 | 3.46 | 0.6-9.36 | 0.27 | 0.02-1.1 |  | 2020 | 20334-7 | UK | 0.27 | 0.05-0.89 | 0.10 | 0.02-0.29 |
| 2021 | 21328-3 | UK | 0.51 | 0.18-1.02 | 0.59 | 0.2-1.24 | 0.73 | 0.27-1.43 | 0.23 | 0.08-0.51 |  | 2021 | 21328-2 | UK | 0.39 | 0.01-1.5 | 0.10 | 0.01-0.33 |
|  |  |  |  |  |  |  |  |  |  |  |  | 2021 | 21328-3 | UK | 0.57 | 0.09-1.95 | 0.10 | 0.03-0.26 |
